# Community Citizen Science for Risk Management of a Spontaneously Combusting Coal‐Mine Waste Heap in Ban Chaung, Dawei District, Myanmar

**DOI:** 10.1029/2020GH000249

**Published:** 2020-06-01

**Authors:** Tanapon Phenrat

**Affiliations:** ^1^ Research Unit for Integrated Natural Resources Remediation and Reclamation (IN3R), Department of Civil Engineering, Faculty of Engineering Naresuan University Phitsanulok Thailand; ^2^ Center of Excellence for Sustainability of Health, Environment and Industry (SHEI), Faculty of Engineering Naresuan University Phitsanulok Thailand

**Keywords:** spontaneous combustion, coal‐mine waste, community citizen science, remedial selection, public participation

## Abstract

Since 2015, a large heap of improperly disposed coal‐mine waste in Ban Chaung, Dawei district, Myanmar, has repeatedly spontaneously combusted, affecting an indigenous community. Recently, the regional Myanmar government has compelled the mine to properly manage the mine waste heap, but there is no opportunity for affected villagers to participate. This study empowers the affected villagers to make risk management decisions via a community citizen science approach. First, field investigations were performed with the affected community to identify hot spots at the waste heap releasing gaseous pollutants that may exceed acceptable levels. Next, existing monitoring data previously collected by the community were interpreted as clear evidence of past poor waste management. Information about suppression of existing fire and mine waste storage options was presented to the community for them to make an informed decision about the most appropriate corrective action that should be taken by the mine. The mining company chose to use surface sealing for both suppression of existing fire and on‐site storage of the mine waste but did not install any long‐term monitoring system. Nevertheless, the community's choice was surface sealing with preventive monitoring together with emergency response, which is the more scientifically appropriate option. This outcome of a science‐based risk management decision by the community will be forwarded to the regional government for enforcement. This process of community citizen science is in line with the normative rationale of public participation, which is meant to influence decisions, elevate democratic capacity, and empower marginalized individuals and communities.

## Key Points

- Spontaneously combusting coal‐mine waste is adversely affecting an indigenous community in Myanmar since 2015
- Community citizen science allowed the affected villagers to make a decision about the most appropriate corrective action
- The community's choice of surface sealing with preventive monitoring is more scientifically appropriate than the mine's choice

## 1 Introduction

The spontaneous combustion of coal and coal‐mine waste during mining, storage, and transportation has been a vexing safety problem for coal producers as well as users for decades (Onifade & Genc, 2019; Song & Kuenzer, 2014; Stracher et al., 2010, 2015). Although coal is stable underground, the exposure of coal and coal‐mine waste to oxygen in the air during and after mining may result in oxidation, an exothermic reaction, and eventually, spontaneous combustion (Dang et al., 2016). As explained by an experimentally proven coal‐oxygen compound theory (Kong et al., 2017; Wen et al., 2017; Zhu et al., 2018), the percolation of air through coal and the coal‐waste heap results in a series of physical adsorption, chemical absorption, and chemical reaction steps, forming oxycoal (equations (1) to (3)) and heat release (Δ*H*). Next, the heat generated causes a further increase in the temperature of the coal, which accelerates the rate of coal oxidation. Eventually, if not properly ventilated, these actions cause a coal or coal‐waste heap to accumulate heat to a critical temperature (55–70°C, so‐called zero activation energy, Zhu et al., 2018), which results in smoldering and eventually, spontaneous combustion (equations (4) and (5)) (Zhang, 2013). Generally, three components are needed to cause a fire: heat, fuel, and oxygen. For spontaneous combustion, coal is the fuel, while the interaction of coal and oxygen yields the heat.

(1) $$\text{Coal}+O_{2}\to O_{2}\;\left(\text{physically sorbed}\right),$$

(2) $$O_{2}\;\left(\text{physically sorbed}\right)\to O_{2}\;\left(\text{chemically sorbed}\right),$$

(3) $$O_{2}\;\left(\text{chemically sorbed}\right)\to \text{surface oxides}\;\left(\text{oxycoal}\right)+∆H,$$

(4) $$\text{surface oxides}\;\left(\text{oxycoal}\right)\to CO_{2}+H_{2}O+∆H;\text{major reaction},$$

(5) $$\text{surface oxides}\;\left(\text{oxycoal}\right)\to H_{2}+\mathrm{CO}+\text{VOCs};\text{side reactions}.$$

Furthermore, coal and coal‐mine waste typically contain pyrite (FeS_2_), which plays a significant role in spontaneous combustion, according to pyrite theory (Deng et al., 2015). Since pyrite oxidation by interaction with oxygen alone (equation (6)) or together with water (equation (7)) is exothermic (Lui et al., 1998; Yang et al., 2019), the released heat catalyzes coal self‐heating and spontaneous combustion, as discussed above. A recent study revealed that a pyrite content of 5–7% in coal has the greatest effect on spontaneous coal combustion (Deng et al., 2015).

(6) $$4\mathrm{Fe}S_{2}+11O_{2}\to 2{\mathrm{Fe}}_{2}O_{3}+8{\mathrm{SO}}_{2}↑+∆H\;\left(3,412\;\mathrm{kj}\;/\mathrm{mol}\right),$$

(7) $$2\mathrm{Fe}S_{2}+7O_{2}+16H_{2}O\to 2\mathrm{FeS}O_{4}\cdot 7H_{2}O+2H_{2}{\mathrm{SO}}_{4}+∆H\;\left(1,321\;\mathrm{kj}\;/\mathrm{mol}\right).$$

As shown in equations (4)–(6), the gaseous pollutants released as a result of the self‐heating, smoldering, and spontaneous combustion of coal and coal‐mine waste are CO_2_ (major greenhouse gas), CO, SO_2_, and volatile organic compounds (VOCs) including benzene, toluene, dichloromethane, and methyl chloride, which are toxic (Guo, Wen, et al., 2019; Pone et al., 2007). Moreover, as coal typically contains other sulfur‐ and nitrogen‐containing organic compounds or minerals, other toxic gases including H_2_S, CS_2_, and HCN are generated (Lui et al., 1998; Pone et al., 2007). Finally, pyrite typically contains a high concentration of toxic metals, and toxic metals and particulate matter are released as a result of this combustion (Pone et al., 2007).

In addition to coal producers, unfortunately, the spontaneous combustion of coal‐mine waste also adversely affects nearby communities by emitting toxic air pollutants (spontaneous combustion of 1 ton of coal and coal‐mine waste produces 0.84‐kg SO_2_, 0.61‐kg H_2_S, 0.03‐kg NOx, 99.7‐kg CO, and 0.45‐kg smoke, Lui et al., 1998). Unsurprisingly, authors of a recent intensive literature review concluded that spontaneous combustion of coal and coal waste is likely to have short‐term adverse respiratory impacts (Melody & Johnston, 2015). Similarly, adverse cardiovascular impacts and increased mortality are also possible, depending on the proximity of the source and the magnitude of exposure to these toxic gaseous pollutants (Melody & Johnston, 2015). Also, oxidation of pyrite in coal and coal waste heaps causes acid mine drainage (AMD), releasing sulfuric acid (equation (7)), which causes degradation of soil and water quality (Núñez‐Gómez et al., 2020). Similarly, spontaneous combustion of metal‐rich coal and coal‐mine waste leaves behind metal‐rich combustion residuals resulting in soil and water contamination (Ribeiro et al., 2010). Unsurprisingly, soil and water contamination occurring as a result of an improperly managed or abandoned coal‐mine waste heap have been reported (Li et al., 2018; Sahoo et al., 2016; Stracher et al., 2015). For this reason, coal‐mine waste requires proper management protocols, including self‐heating predictions, preventive monitoring and storage, fire detection, and emergency response for fire control to minimize spontaneous combustion and avoid unacceptable risks (Onifade & Genc, 2019; Sloss, 2015).

In Ban Chaung in the Dawei district of Tanintharyi region, southern Myanmar, an indigenous Karen community has faced health and safety risks due to a large heap of improperly managed coal‐mine waste that has been spontaneously combusting intermittently since 2015. While in 2017, the regional Myanmar government ordered the mine to improve the management of the mine waste heap to stop and prevent further spontaneous combustion, the mine responded by covering the waste heap with soil and gravel, which has proven to be insufficient to achieve the goal. Despite the suspension of the mine since 2017, the community is still at risk, as the coal‐mine waste continues to burn, releasing toxic fumes. Furthermore, the community lives in fear and uncertainty that the spontaneous combustion may trigger wildfire, since the waste heap is in the middle of a richly forested area.

Recently, the regional Myanmar government has again demanded that the mining company properly manage the spontaneous combustion as well as the mine waste heap, but the affected villagers have not been informed about the mining company's management approach and thus are not sure whether this round of corrective action will fail again. Consequently, the objective of this study is to empower the affected villagers to participate in risk management decision‐making via a community citizen science approach, defined by US EPA as “collaboratively led scientific investigation and exploration to address community defined questions, allowing for engagement in the entirety of the scientific process” (National Advisory Council for Environmental Policy and Technology, 2016). Citizen science and community citizen science have been effectively used in several environmental and public health protection projects (Averett, 2017; Breuer et al., 2015; Cabanellas‐Reboredo et al., 2019; Fritz et al., 2019). US EPA elaborates that community citizen science may include partnerships with professional scientists but is oriented toward community goals and working together in scalable networks to encourage collaborative learning and civic engagement (National Advisory Council for Environmental Policy and Technology, 2016). For this reason, in this study, we worked with the affected community to perform a field investigation of the current mine waste heap, interpret monitoring data that the community had previously collected, use advanced scientific tools to back up the community's data, inform the community about available risk management options for the spontaneous combustion of coal‐mine waste, evaluate those options based on US EPA's nine criteria for remedial option selection (United States Environmental Protection Agency, 1990), and equip the community with sufficient scientific knowledge to make a decision about the most appropriate corrective action for the mining company to take. The outcome is a rational, scientific‐based risk management decision by the community, which will be forwarded to the regional government to enforce against the mine. Thus, this process of community citizen science provides meaningful data for social license to operate (Hitch et al., 2020). Moreover, the process of community citizen science is in line with the normative rationale of public participation, which is meant to influence decisions, elevate democratic capacity, provide social learning, and empower and emancipate marginalized individuals and communities (Otwong & Phenrat, 2017). This study is the first record of field‐scale community citizen science in Myanmar, especially for risk management of mine waste, a significant environmental crisis in Myanmar affecting not only the ecosystem but also quality of life and human rights (Htwe, 2019; Lee, 2019; Nang & Paddock, 2019).

## 2 Methodology

### 2.1 Community Citizen Science Strategy

The main strategy was to establish a research team consisting of both a professional scientist (T. Phenrat) and the local monitoring group, which is made up of volunteers from the affected community. As a community citizen science project, the objective of the research, that is, to allow the affected community to participate in risk management decision‐making for the mine waste heap, was established by the affected community itself during the first meeting (Figure S1 in supporting information [SI]). The overall framework followed the preliminary assessment/site investigation (PA/SI) and feasibility study as well as the remedial selection criteria of the US EPA according to the Comprehensive Environmental Response, Compensation, and Liability Act (CERCLA) (US EPA, 2018). Notably, not all of the steps in CERCLA were implemented in this study; only the relevant steps sufficient to address the objective of this study were performed.

Since the professional scientist and the local monitoring group have different areas of expertise, both of them were involved in project planning, implementation, and interpretation of results. The unique insight and expertise that the local monitoring group brought to the project included (1) a database of affected villagers (amount and their locations), (2) records of previous incidents (historical photos and counts of fire frequency), (3) past environmental monitoring results (past water and air samplings), (4) survey of health impacts, (5) knowledge of the hot spots, seasonal effects on combustion incidents, and locations of impacted agricultural land and surface water, and (6) a channel to voice their concerns and risk management decision to a responsible government agency. On the other hand, the insight and expertise that the professional scientist provided included (1) the use of appropriate field and remote scientific techniques for PA/SI, (2) knowledge of preliminary risk assessment through comparison with relevant guidelines, (3) knowledge of feasible suppression of existing fire and proper mine waste storage options as well as their evaluation criteria, and (4) insight into normative public participation protocols to empower affected communities for informed risk management decision‐making. The detailed procedures are as follows.

### 2.2 Current Site Inspection

The local monitoring group led the field PA/SI by planning the sampling locations at the mine waste heap, the mining area, the mine lake, the affected surface water, and the affected agricultural land (Figure S2 in SI). Two rounds of site inspection were conducted in July and August 2019. We used a handheld thermal infrared camera (Fluke, Model 561) to identify hot spots in the coal‐mine waste. A normal camera was also used to take photos of the waste heap and hot spots. Then three types of portable gas detector were used to measure concentrations of volatile organic compounds (VOCs), hydrogen cyanide (HCN), ammonia (NH_3_), and hydrogen sulfide (H_2_S) (RAE, Model MultiRAE Lite), lower explosive limit (LEL), oxygen (O_2_), nitrogen dioxide (NO_2_), nitrogen oxide (NO) and sulfur dioxide (SO_2_) (Industrial Scientific, Model IBRID Mix6), and carbon monoxide (CO) (Smart Sensor, Model AS8900) in the open space right next to the hot spots. At each station, we measured for 20 min and reported the maximum concentrations measured during the 20‐min period. The same measurements were made at the open space on the top of the waste heap, at the base of the waste heap, at the mine lake, and at the affected agricultural land, as well as in residential areas far from the waste heap to provide a preliminary evaluation of gaseous pollutants at different distances from the source. Composite coal and coal‐mine waste samples were collected from the mine and the waste heap. Also, composite soil samples were collected from affected agricultural land and unaffected residential land. The metal and metalloid concentrations of the composite coal, coal waste, and soil samples were measured by X‐ray fluorescence (XRF) (Olympus, Model DS‐6500‐C Delta series). Water samples were collected from the mine lake, a highly affected surface water body, and a moderately affected surface water body. The values of pH and conductivity were measured on site. An unaffected area was selected as a control area for reference measurements of temperature, air quality, and water quality. Figure S3 in SI depicts the sampling locations, while Table S1 summarizes their description. A GPS device was used to collect coordinates of the boundaries of the waste heap. The shape of the waste heap was constructed while its volume was calculated.

### 2.3 Community's Previous Monitoring Data and Landsat 8 for Elevated Temperature Anomaly Identification

Previous data collected by the local monitoring group included photos of the mine waste heap smoldering in 2015, ambient air quality results in an open space at the base of the mine waste heap in 2018 (sampled and analyzed by Ecolab, an accredited laboratory service in Myanmar), and daily records of smoldering and spontaneous combustion incidents from 2017 to 2018. These data were reviewed and analyzed in this study as evidence of poor past management of the mine waste heap. Moreover, surface temperatures in the vicinity of the mine and the mine waste heap collected by the Thermal Infrared Sensor (TIRS) of the Landsat 8 satellite from 2013 to 2018 were used to determine surface temperature anomalies as an indicator of spontaneous combustion of the coal and coal‐mine waste. This was also taken as evidence of the poor past management practices applied to the mine waste heap.

### 2.4 Community Empowerment and Participatory Risk Management Decision

Available options for fire suppression and proper storage of mine waste to prevent further spontaneous combustion and environmental contamination were reviewed and evaluated based on seven of US EPA's nine criteria for remedial option selection (United States Environmental Protection Agency, 1990). While the nine criteria consist of (1) overall protection of human health and the environment, (2) compliance with applicable or relevant and appropriate requirements, (3)long‐term effectiveness and permanence, (4) reduction of toxicity, mobility, or volume through treatment, (5)short‐term effectiveness, (6) implementability, (7) cost, (8) community acceptance, and (9) state acceptance, only the first seven criteria were evaluated in this first step (see the scoring system in Table S2 in SI). Then, in November 2019, we presented this information to the community in order for them to have sufficient scientific knowledge of these options and evaluations. Next, we allowed the affected community to assign a score to each option to determine their most preferable options. The scores they assigned to each option represented the eighth criterion and were used to calculate the total score based on the first eight of the nine criteria of remedial option selection. This process allowed the community to make an informed decision and be a part of systematic risk management decision‐making about the most appropriate corrective action that should be taken by the mine. The ranking of management options as well as the community's preference will be passed on to the relevant government agencies.

## 3 Results and Discussion

### 3.1 Current Spontaneous Combustion at the Coal‐Mine Waste Heap and Release of Toxic Gaseous Pollutants

**Figure 1:**
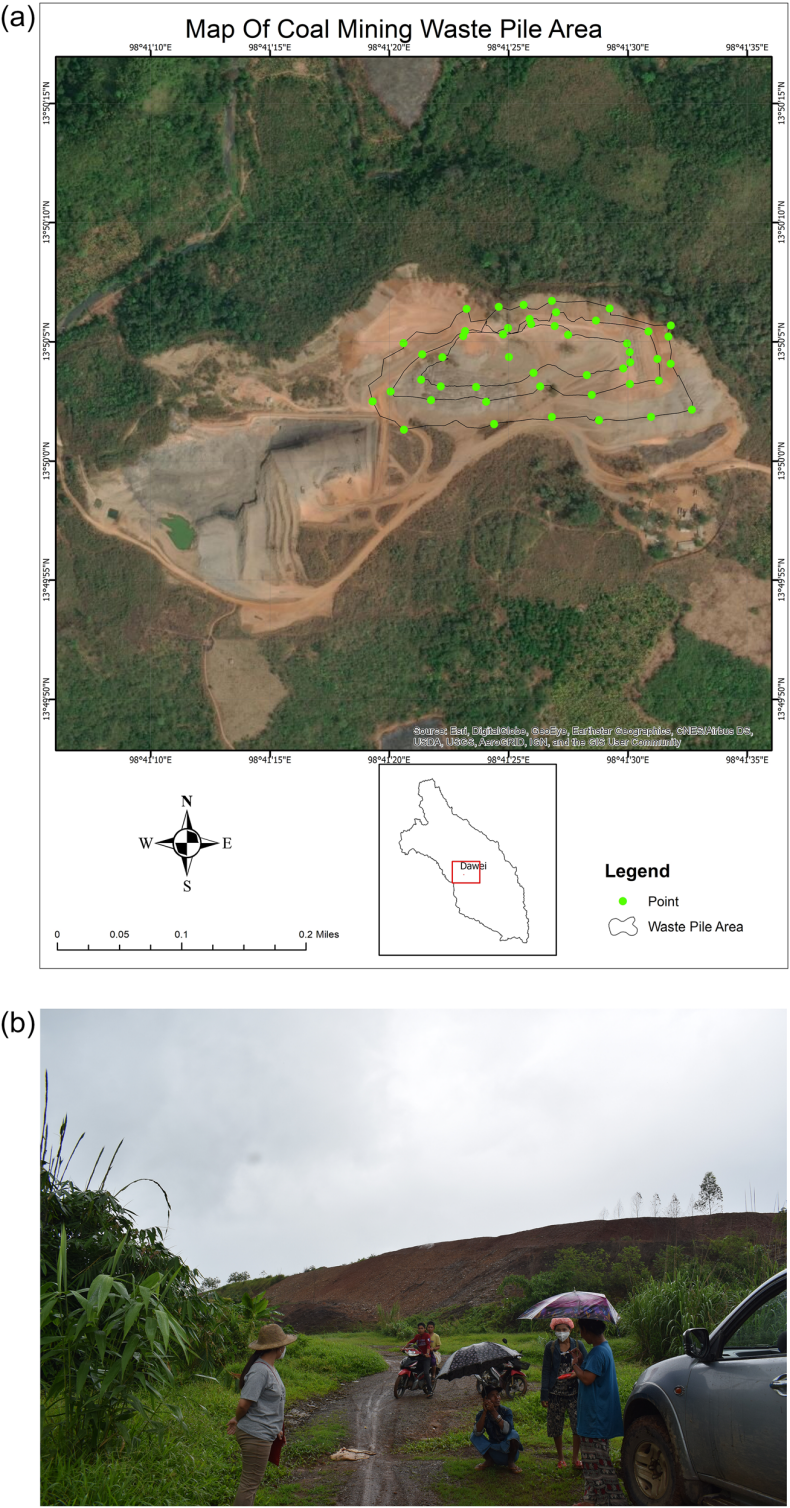
(a) Top view of mine waste heap, mining area, and mine lake (taken in 2018) and (b) side view of the mine waste heap (taken in 2019).

Currently, the coal‐mine waste heap has an approximate footprint of 50,400 m^2^ (5.04 ha) and a height of 26.88 m as determined from the GPS coordinates collected in the field (see Figure 1). Thus, its approximate volume is 885,296 m^3^, which roughly corresponds to 1,284,584 tons of mining waste mixed with the soil that covers the waste heap. We observed several areas where the soil layer does not sufficiently cover the coal waste as well as some areas where bare coal waste is piled up without any covering soil layer at all. Moreover, several cracks and holes are visible on the surface of the large coal waste heap. These three aspects of improper management release substantial toxic gases to the atmosphere. Some of them were measured as shown in Table 1.

**TABLE 1:** Concentrations of Toxic Gaseous Pollutants at the Mine Waste Heap as Well as in the Residential Areas and the Control Area Measured During the Field PA/SI in 2019 and Previously Sampled and Analyzed by ECOLAB on Behalf of the Local Monitoring Group in 2018

No.	Description	Location	LEL	O_2_ (%)	Concentration (ppm)							
					CO	NO_2_	SO_2_	NO	VOC	H_2_S	NH_3_	HCN
1	Control	Control area	—	20.8	—	—	—	—	—	—	—	—
2	Part of waste heap with insufficient soil cover	At the base of the waste heap	—	20.4	35	—	—	—	33	—	1	—
3	A crack	On top of the waste heap	—	19.5	60	—	0.2	3	168	1.2	3	0.5
4	A crack	On top of the waste heap	—	15.6	300	—	17.9	4	234	3.6	2	3.0
5	A small waste pile in open space	In the middle layer of the waste heap	—	20.9	—	—	0.2	3	81	0.6	—	1.5
6	Open space	On top of the waste heap	—	21.2	—	—	2	—	43	—	—	—
7	Mine lake	Close to the coal mine		20.9	—	—	—	—	107	—	—	1
8	Open space	0.1 km from the waste heap	—	20.7	—	—	—	—	9	—	—	—
9	A residential area	0.5 km from the waste heap	—	21.0	—	—	—	—	4	—	—	—
10	Playground	A school 1.2 km from the waste heap	—	20.6	—	—	—	—	5	—	—	—
11	A residential area	1.3 km from the waste heap	—	20.5	—	—	—	—	2	—	—	—
12	2018 results by villagers^d^	0.1 km from the waste heap	NA	NA	0.38	0.1	0.16	NA	19.39 (hydrocarbon)	NA	NA	NA
		Ambient air quality guidelines (US EPA, 2017)	—	—	35^a^/9^b^	0.1^a^	0.19^c^/0.14^a^/0.075^d^	—	1–3 medium 3–10 poor >10 bad	0.01^d^	0.14^d^	0.31^a^
					Headache, dizziness, vomiting, and nausea	Acute bronchitis, dyspnea, cyanosis, chest pain, rales, headaches, eye irritation, a dry nonproductive cough, and vomiting	Irritating to the eyes, mucous membranes, skin, and respiratory tract		Malodor, irritations of the nose, throat, and eyes, cause headaches, nausea, dizziness, allergic skin reactions	Malodor (rotten egg smell)	Coughing, and nose and throat irritation	Non‐fatal effect including rapid breathing, vomiting, and a feeling of suffocation

*Note*. NA, not analyzed; —, lower than detection limit.

^a^ Hour average concentration.

^b^ Eight‐hour average concentration.

^c^ Ten‐minute average concentration.

^d^ Twenty‐four‐hour average concentration.

**Figure 2:**
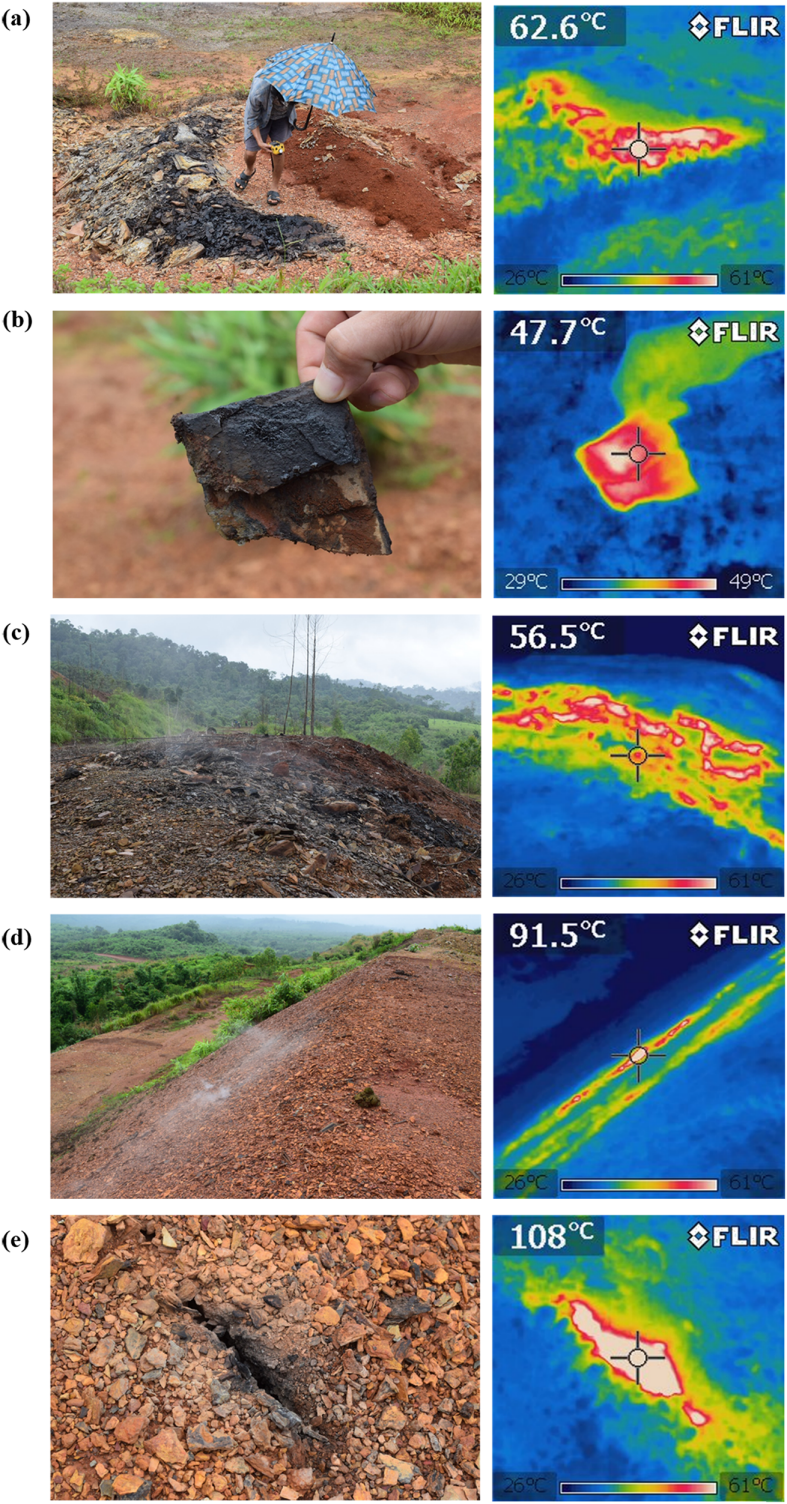
(a) and (b)Low‐temperature oxidation of an abandoned small waste pile. (c) Smoldering of the mine waste with insufficient soil cover. (d) Smoldering of the mine waste with insufficient soil cover (presumably due to erosion of the soil cover layer). (e) Spontaneous combustion inside of the large mine waste heap that results in the formation of cracks, releasing vapor and toxic emissions.

Even in 2019, the coal‐mine waste heap was still spontaneously combusting. All phenomena associated with spontaneous combustion, including low‐temperature oxidation, smoldering, and combustion, were observed during the site investigation. Figure 2a presents a pair of normal and thermal IR images of coal‐mine waste improperly dumped in an open space on top of the large waste heap. The maximum temperature of this small waste pile was 62.6°C, which is in the temperature range for zero activation energy (55°C to 70°C). Here, we observed the beginning of the smoldering process. At the same time, some pieces of mine waste in this small heap were still in the low‐temperature oxidation process (see an example in Figure 2b where the temperature was 47.7°C, lower than the critical temperature for zero activation energy). Thus, no smoke was observed from this piece of coal waste. Nevertheless, both low‐temperature oxidation and smoldering release toxic gaseous pollutants. The maximum ambient concentrations of pollutants measured close to the small mine waste pile were 81 ppm for VOCs, 0.6 ppm H_2_S, 1.5 ppm HCN, 0.2 ppm SO_2_, and 3 ppm NO. In contrast, these gases were non‐detectable in a control area far away from the waste heap. Thus, even a small abandoned waste pile can release a substantial concentration of toxic pollutants.

Smoldering and release of toxic gases also occurred in areas on the large waste heap where soil cover was not intact. As shown in Figure 2c, the temperature of the coal‐mine waste exposed to the air was 56.6°C, while that of the area with intact soil cover was 26°C to 29°C. Similar to that of the small waste pile, as a result of smoldering, the maximum ambient concentrations of pollutants measured close to this point were 33 ppm for VOCs, 35 ppm CO, and 1 ppm NH_3_. Similar to Figure 2c, smoldering as a result of insufficient soil covering also occurred in Figure 2d, which appeared to be more severe.

Spontaneous combustion also occurred internally, deep inside the waste heap. Figure 2e shows a crack on top of the waste heap, in which the temperature was as high as 108°C. The maximum ambient concentrations of pollutants measured close to this point were 234 ppm for VOCs, 300 ppm CO, 17.9 ppm SO_2_, 4 ppm NO, 3.6 ppm H_2_S, 2 ppm NH_3_, and 3 ppm HCN. The internal spontaneous combustion of the coal‐mine waste heap as well as the formation of cracks can be explained by coal‐oxygen compound theory (Sasaki & Sugai, 2011; Zhu et al., 2018). First, due to the poor soil coverage discussed above, oxygen from the atmosphere can still interact with the outside surface of the coal‐mine waste heap and that oxygen diffuses inward to the center of the heap (Guo, Yan, et al., 2019; Wen et al., 2017). The oxidized outer part and the inner part of the heap generate heat, some of which is dissipated to the atmosphere after enough time, while the rest of the heat may radiate deeper into the heap. When the coal‐mine waste at the center of the heap is preheated slowly without oxygen, a high temperature spot is generated at the center. The oxidation and heat generation zone gradually moves from the waste heap surface to the center while shrinking and increasing in temperature. Eventually, a hot spot is formed at the center, and oxygen diffuses to the center region, causing spontaneous combustion. The hot spot is projected preferentially through a highly permeable path where oxygen is rich, toward the mine waste heap surface. This eventually forms cracks on the surface of the coal waste heap, releasing toxic gases, as observed in Figure 2e. Several other cracks were observed on the large waste heap. Notably, a substantial amount of hot gas was released through these cracks.

Close to the locations of low‐temperature oxidation, smoldering, and spontaneous combustion, the measured air pollutants (maximum concentrations of 20‐min measurement) exceeded the acceptable levels (either 1‐hr averaged or 8‐hr averaged concentration) according to the US EPA ambient air quality standard (US EPA, 2017) (Table 1). For example, observed CO concentrations ranged from 35 to 300 ppm, while the acceptable levels are 9 and 35 ppm for 8‐hr averaged and 1‐hr averaged concentrations, respectively (US EPA, 2017). This trend was also true for SO_2_, H_2_S, VOCs, NH_3_, and HCN. The VOCs appeared to be the most severe, as the measured VOC concentrations at the waste heap were from 33 to 234 ppm, whereas a guideline states that VOCs >10 ppm is classified as bad air quality (Meyer, 2018). Similarly, the measured H_2_S concentrations (0.6 to 3.6 ppm) were much higher than the acceptable level (0.01 ppm) (US EPA, 2017). This suggests that the air pollutants from the coal‐mine waste heap can pose health risks to villagers who approach or work near the heap. The possible symptoms are also summarized in Table 1. Interestingly, several of the symptoms in Table 1, including irritations of the nose, throat, and eyes, headaches, nausea, dizziness, and allergic skin reactions, are in good agreement with the villagers' complaints about the effects of air pollution from the waste heap on their health. It should be noted that although this study measured toxic gaseous concentrations for only 20 min and reported the maximum concentrations of each pollutant at each location, these values can be taken as preliminary indicators that the pollutants released from this coal‐mine waste heap potentially pose serious health risks. Nevertheless, further detailed analysis (1‐hr averaged, 8‐hr averaged, and 24‐hr averaged concentrations) should be performed for a full analysis and comparison with guideline values. Notably, in this study, the monitoring results were compared with the ambient air quality standards of the US EPA because Myanmar has not established comprehensive ambient air quality standards, only emission guidelines for NO_2_, ozone, particulate matter, and SO_2_ (Ministry of Natural Resources and Environmental Conservation, 2015). The Myanmar guidelines neither extend to all relevant toxic gaseous pollutants nor are appropriate for the type of ambient exposure described within this study.

Although this study measured several main air pollutants, as reported in Table 1, no measurements for metal or metalloid fumes were conducted due to limitations of equipment and logistics. Nevertheless, considering the elevated metal and metalloid concentrations (especially arsenic and mercury) in the coal‐mine waste samples reported in Table 2, toxic metals and metalloids would be expected to be released together with other air pollutants, which worsens health risks to the nearby villagers even more. As a result, to effectively assess and manage the health risks posed by the heap, further appropriate quantification of metal and metalloid concentrations (especially arsenic and mercury) in the pollutant fumes released from the heap is highly recommended.

**TABLE 2:** Metal and Metalloid Concentrations in Coal Samples and Coal‐Mine Waste Samples as Well as Affected and Non‐affected Soil Samples as Measured by XRF

Sample	Concentration (mg/kg)											
	As	Ba	Cd	Cr	Cu	Fe	Hg	Mn	Ni	Pb	S	Zn
Coal	—	350.5 ± 6.4	—	32.0 ± 1.4	20.5 ± 0.7	2,799.0 ± 36.8	—	42.0 ± 4.1	—	78.0 ± 1.4	1,995.5 ± 310.4	8.4 ± 6.2
Coal waste from the mining site	18.0 ± 5.6	442.5 ± 20.5	—	58.0 ± 5.7	16.0 ± 2.8	41,038.0 ± 736.8	0.9 ± 1.3	199.0 ± 5.7	27.0 ± 1.4	49.0 ± 1.4	5,512.5 ± 279.3	34.0 ± 0.0
Coal waste on the heap	52.5 ± 0.7	600.5 ± 7.8	—	81.0 ± 8.5	20.0 ± 5.7	52,738.0 ± 1,637.7	2.1 ± 0.2	1,500.0 ± 131.5	—	46.5 ± 0.7	9,036.5 ± 1,236.7	70.5 ± 16.2
Coal waste soil on the heap	49.0 ± 0.0	552.0 ± 60.8	—	79.5 ± 3.5	11.5 ± 11.3	58,876.0 ± 1,312.4	0.8 ± 1.2	702.0 ± 151.3	—	48.5 ± 0.7	2,978.5 ± 522.6	29.0 ± 1.4
Affected agricultural soil close to the waste heap	11.5 ± 0.7	408.0 ± 45.2	—	50.0 ± 5.7	—	43,266.5 ± 2,895.6	—	675.5 ± 9.2	15.5 ± 21.9	27.0 ± 5.7	—	24.0 ± 11.3
Non‐affected agricultural soil	3.8 ± 5.4	367.5 ± 50.2	—	54.5 ± 3.5	19.5 ± 0.7	18,943.0 ± 567.1	—	454.0 ± 7.1	30.5 ± 14.8	20.5 ± 5.0	—	25.0 ± 1.4

### 3.2 Current Situation of Affected Environment

The gaseous pollutants released from low‐temperature oxidation, smoldering, and spontaneous combustion were dispersed and diluted by the wind. These processes both attenuate and migrate the pollutants. However, as shown in Table 1, VOCs were the only pollutant that was still detected in all locations measured in this study, suggesting that all other pollutants were attenuated during migration. VOC concentrations at a mine lake near the mine and mine waste heap, on top of the waste heap, and at the base of the waste heap were 107, 43, and 9 ppm, respectively. The high VOC concentration at the mine lake may be the result of a downwash effect due the high elevation of the mine waste heap and low elevation of the adjacent mine lake area. According to the guideline (Meyer, 2018), the air quality at the top and the base of the waste heap was bad, while the air quality at the mine lake was very bad. Moreover, VOC concentrations at a villager's house (0.5 km from the waste heap) and the playground of a nearby school (1.2 km from the waste heap) were 4 and 5 ppm, respectively, which may be classified as poor air quality according to the guideline (Meyer, 2018). Finally, the VOC concentrations at another resident's house (1.3 km from the waste heap) and a control area (12 km from the waste heap) decreased to 2 and 0 ppm, respectively.

**Figure 3:**
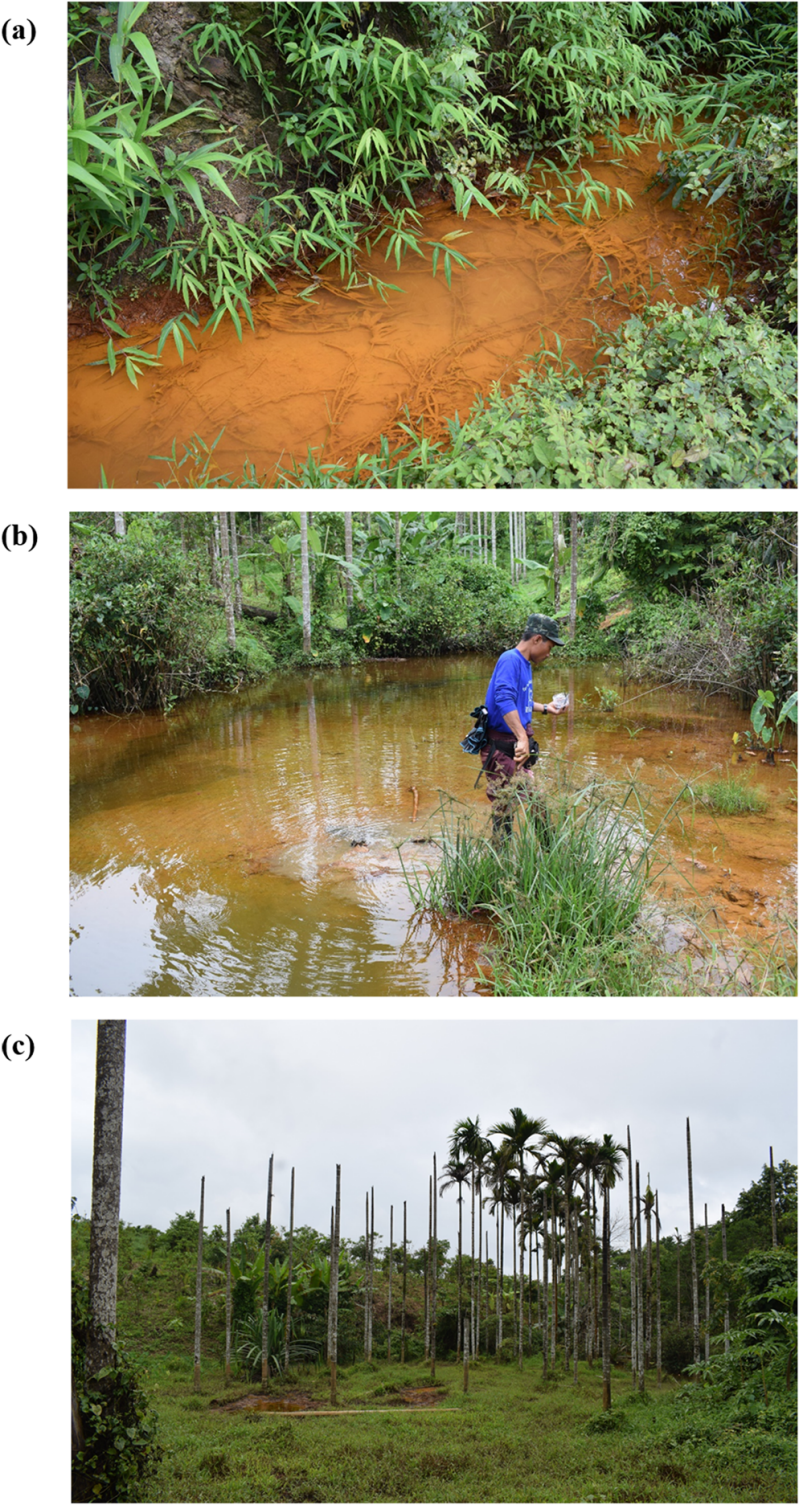
(a) Affected surface water, (b) affected agricultural land, and (c) dead betel nut trees.

In addition to air pollution, surface water and surface soil contamination was also observed. As shown in Figures 3a and 3b, rusty precipitate, a sign of acid mine drainage, was observed in natural surface water channels close to the mine waste heap. The pH values of the affected surface water ranged from 2.67 to 3.17, and the electrical conductivity values were 1,500 μS/cm while the pH and electrical conductivity of a water sample from a control area were 7.42 and 600 μS/cm, respectively. The acid mine drainage observed here is consistent with the high sulfur content of the mine waste (Table 2) and with the fact that no water management system to divert mine water and mine drainage was observed at the waste heap. The acid mine drainage had also affected an agricultural area nearby. Presumably due to low pH and high electrical conductivity, betel nut trees, a plant with low salt tolerance (Staples & Bevacqua, 2006), were dead (Figure 3c). The affected agricultural soil had substantially higher arsenic and iron concentrations than the soil sample from an unaffected control area (Table 2). The arsenic and iron contaminations are clear signatures of acid mine drainage contamination. No remediation of either the affected natural waterway or the affected agricultural soil has been performed.

### 3.3 Evidence of Poor Past Management of the Mine Waste Heap

**Figure 4:**
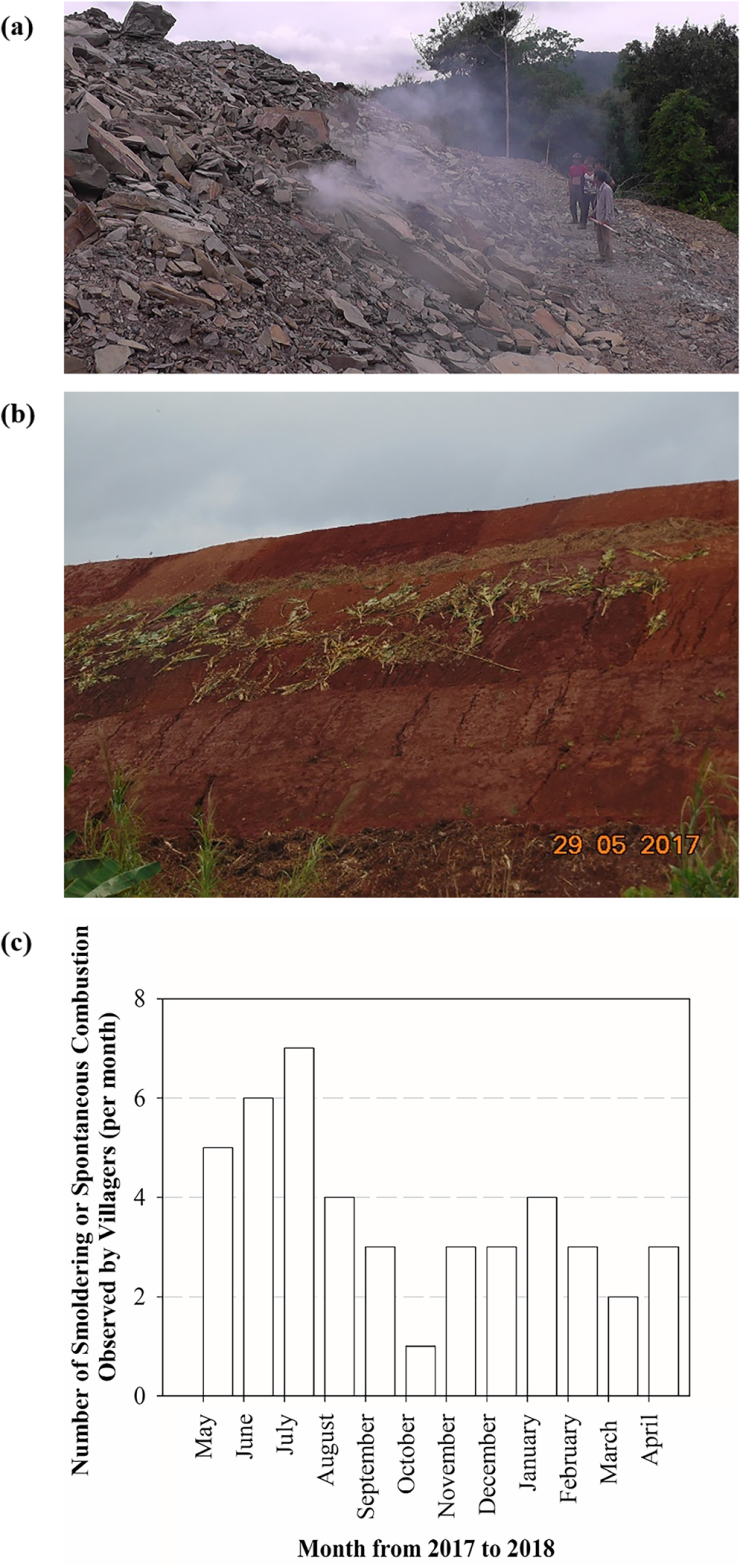
(a) A photo showing poor past management of the mine waste heap, as smoldering was occurring and there was no soil layer cover. (b) Past corrective action of the mine by placing a soil layer and banana leaves on the waste heap to prevent spontaneous combustion. (c) The monthly frequency of observable smoldering or spontaneous combustion from 2017 to 2018 as recorded by the local monitoring group.

Although the professional scientist on this project (T. Phenrat) was only able to investigate the spontaneous combustion incident in 2019, several forms of evidence of poor past management of the mine waste heap leading to spontaneous combustion were recorded by the affected villagers. As shown in Figure 4a, based on a photo taken in April 2015, smoldering also occurred at the poorly managed waste heap at that time, releasing toxic gases to the atmosphere. No soil covering was observed in the photo, suggesting that the management of the mine waste heap in 2015 was much poorer than the current condition. For this reason, the past impacts would have been even more.

In 2017, when the regional Myanmar government ordered the mining company to improve the management of the mine waste heap to stop the ongoing spontaneous combustion and prevent further incidents, the mining company responded by placing a soil layer and banana leaves on the waste heap (Figure 4b). It could be observed that the soil layer was not well compacted and began to erode not long after it was put in place. Furthermore, neither an appropriate monitoring system (see examples in SI) nor an emergency response protocol for spontaneous combustion is in place. Consequently, spontaneous combustion occurs continuously, proving that this corrective action has been ineffective. Moreover, the local monitoring group recorded the monthly frequency of smoldering or spontaneous combustion, as shown in Figure 4c, an example of the frequency records from 2017 to 2018. In total, there were 47 occurrences of observable smoldering or spontaneous combustion observed by villagers from May 2017 to May 2018, an average of 3.6 times per month. This does not include spontaneous combustion inside the waste heap, which cannot be observed with the bare eye. Notably, the spontaneous combustion incidents were the most frequent during the rainy season (May to August), when up to seven incidents took place per month. This is consistent with the role of water (if less than 40% to 50%) in spontaneous combustion enhancement (Zhu et al., 2018).

In addition, the villagers had managed to have Ecolab, a certified laboratory service in Myanmar, collect an ambient air sample in 2018 at the base of the waste heap. The results, summarized in Table 1, reveal that the air quality was poor with respect to VOC concentration (19.39 ppm), while the other pollutants, such as SO_2_ and NO_2_, were on the edge of the maximum acceptable levels. The high VOC concentration agreed well with the new measurement reported in the previous section. The villagers also had Ecolab monitor the acidity of the affected surface water from 2015 to 2017 and found that the pH values of the affected creek ranged from 3.1 to 3.3, while the pH values of an unaffected creek were neutral, that is, pH from 6 to 7.7. This confirms the occurrence of acid mine drainage from 2015 to the present.

**Figure 5:**
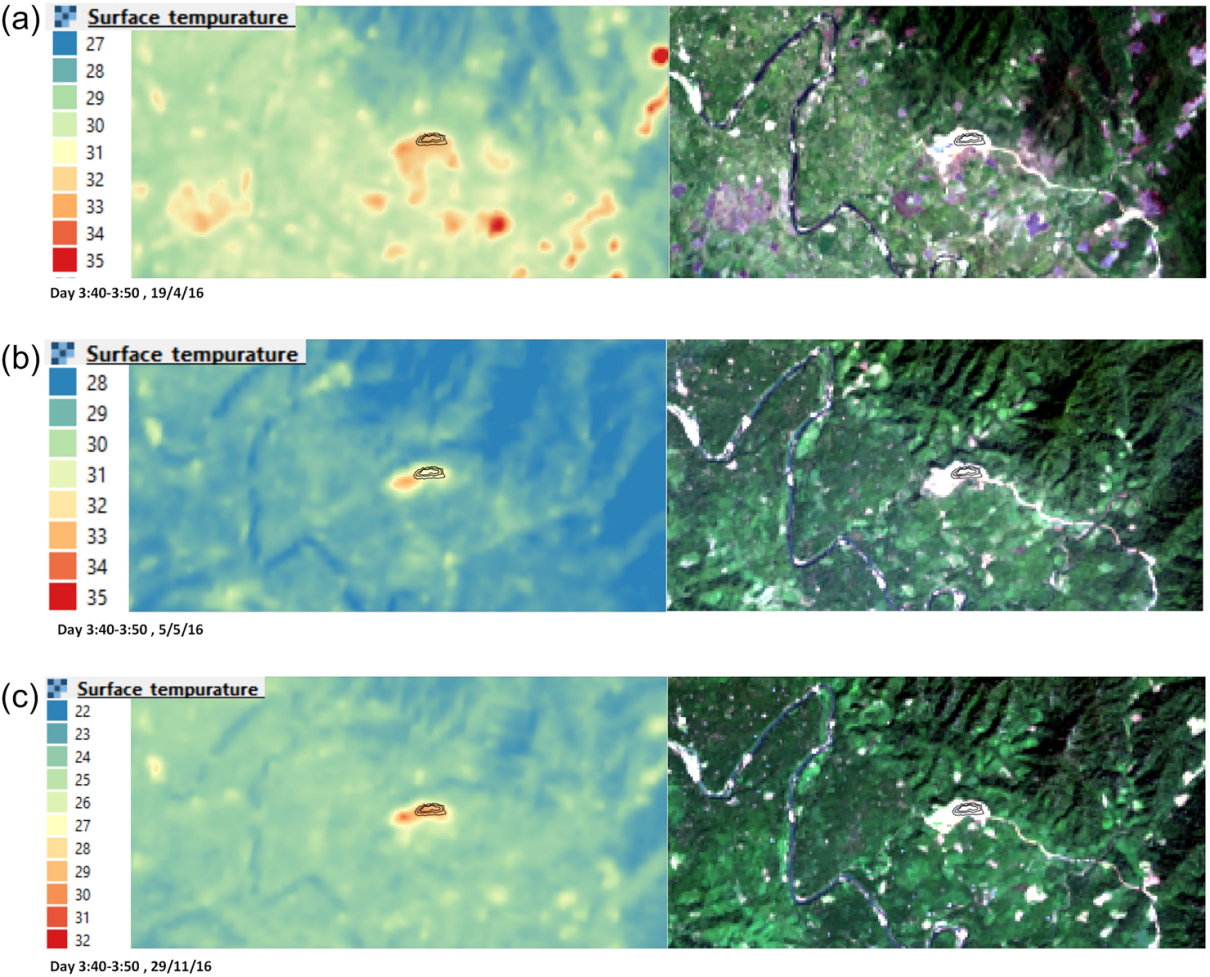
Surface temperatures observed by Landsat‐8 TIRS in 2016 during (a) summer, (b) monsoon, and (c) winter. The polygon represents the location of the mine waste heap.

Along with the field data, Landsat‐8 TIRS observations were used to confirm poor past management of the mine waste heap through a hot spot analysis of imagery collected from 2013 to 2019. As shown in Figure 5 (and in Figures S4 to S9 in SI), in which the polygon is the location of the heap, the coal‐mine waste heap has been a clear and permanent hot spot (red color, which suggests an elevated temperature zone) since 2014. It should be noted that the coal‐mine waste heap and the open coal mining area are the only two permanent hot spots for every season since 2014. Obviously, in the summer of every year there are a lot of hot spots, but most are temporary and correspond to open biomass burning or forest fire spots because they appear only in the summer, not in the winter or the monsoon season. These are distinct from the sites of spontaneous combustion that occurs every season.

As shown in the 2013 imagery (Figure S4 in SI), when no mine waste heap existed, the open mining area was the only hot spot due to the interaction between the open surface of the coal vein and coal seam with oxygen in the air. The same physicochemical phenomena described previously for coal waste and oxygen are also applicable for the coal vein and coal seam, resulting in low‐temperature oxidation, smoldering, or even spontaneous combustion, all of which forms a hot spot. Nevertheless, in 2013, when the mining was in the beginning phase, the hot spot by the open coal mining area occurred only in the summer and the winter, not in the monsoon season. Presumably, in the monsoon season, spontaneous combustion in the coal vein and coal seam was suppressed by infiltrated rainwater that exceeded 40% to 50% (Zhu et al., 2018).

From 2014 on, in every season (Figures S5 to S9 in SI and Figure 5), a permanent hot spot has been present at the coal‐mine waste heap. The surface temperature elevation of approximately 5°C to 10°C at the mine‐waste heap hot spot is consistent with the underground spontaneous combustion observed in other cases (Nguyen & Vu, 2019; Saraf et al., 1995). This confirms that spontaneous combustion inside the waste heap as well as low‐temperature oxidation and occasional smoldering on the surface of the waste heap have been ongoing since 2014. Thus, the emissions of toxic gaseous pollutants that we detected in our field investigation in 2019 likely occurred in the past as well. Based on this 5‐year hot spot trend, spontaneous combustion is likely to continue in the future, should no action be taken to properly manage the waste heap. Notably, rehabilitation of the open mining area is also needed, because it has been a hot spot since 2014 as well. Although no smoldering or spontaneous combustion is currently observed in the open mining area, low‐temperature oxidation may occur and release toxic air pollutants.

### 3.4 Available Management Options and Their Evaluation Based on US EPA's Criteria for Remedial Selection in Comparison to the Options Being Performed by the Mine

Two types of management strategies are needed to address the spontaneous combustion of this coal‐mine waste heap: (1) suppression of existing fires and (2) proper mine waste storage and prevention of future spontaneous combustion. According to the literature (Leal et al., 2016; Li et al., 2019; Li et al., 2019; Lu et al., 2017; Qin et al., 2016; Querol et al., 2011; Song & Kuenzer, 2014; Stracher et al., 2015; Xi & Li, 2016; Zhai et al., 2017), four options for suppression of existing fire were evaluated: (1) no action, (2) excavation, (3) surface sealing, and (4) grouting or injection of inert gas and inhibitor. On the other hand, for proper mine waste storage and prevention of future spontaneous combustion, five options were evaluated in this study: (1) no action except long‐term preventive monitoring, (2)on‐site storage with surface sealing and long‐term preventive monitoring, (3)on‐site secure landfill and long‐term preventive monitoring, (4)on‐site storage in mine lake and long‐term preventive monitoring, and (5)off‐site management: disposal in a secure landfill or conversion of waste to energy. It should be noted that “no action” is always an option to be evaluated according to US EPA (United States Environmental Protection Agency, 1990). Further explanation and brief descriptions of each technique are available in SI. The scoring criteria and scoring system established for this evaluation are also available in the SI (Tables S2 to S11).

Table 3 summarizes the scores of the options according to the first seven US EPA criteria. It should be noted that Table 3 presents the options for the two management strategies together. It starts with suppression of existing fire followed by proper mine waste storage and prevention of future spontaneous combustion. As shown in Table 3, while suppression of existing fire by excavation and grouting or injection of inert gas and inhibitor can be followed by any of the options for proper mine waste storage and prevention of future spontaneous combustion, it is most logical for fire suppression by surface sealing to be followed by the option of on‐site storage with surface sealing and long‐term preventive monitoring for proper mine waste storage and prevention of future spontaneous combustion. According to the scoring system based on seven criteria and the data available in the literature, surface sealing and grouting or injection of inert gas and inhibitor appeared to be the best two management options for the suppression of existing fire, while off‐site disposal and on‐site secure landfill appeared to be the best two management options for proper mine waste storage and prevention of future spontaneous combustion. The no‐action and on‐site storage options for coal‐mine waste in the mine lake and long‐term monitoring are not considered viable options because both of them scored zero for the first two criteria, that is, (1) overall protection of human health and the environment and (2) compliance with applicable or relevant and appropriate requirements, disqualifying them from being a remedial option according to the US EPA criteria for remedial option selection (United States Environmental Protection Agency, 1990).

**TABLE 3:** The Scores of the Options for the Two Management Strategies, According to the First Seven US EPA Criteria, Combined With the Community Acceptance Score

Suppression of existing fire (score based on 7 criteria with a total possible score of 35), [score based on community with a total possible score of 5]	Mine waste storage and prevention of future spontaneous combustion (score based on 7 criteria with a total possible score of 35), [score based on community with a total possible score of 5]
No action (0), [0]	No action (0), [0]
Excavation (18), [0]	No action and long‐term monitoring (5), [3]
	On‐site secure landfill (28), [5]
	Disposal in the mine lake (0), [0]
	Off‐site disposal (30), [5]
Surface sealing (20), [3]	Surface sealing with monitoring (17), [5]
Grouting or injection of inert gas and inhibitor (28), [5]	No action and long‐term monitoring (5), [3]
	Surface sealing with monitoring (18), [5]
	On‐site secure landfill (28), [5]
	Disposal in the mine lake (0), [0]
	Off‐site disposal (30), [5]

Despite these scores, the options that are currently being performed by the mine without any public participation are surface sealing using sandy clay for suppression of existing fire followed by on‐site storage of the mine waste with the same surface sealing, but without any long‐term preventive monitoring (Figure S20 in SI). Notably, in 2019, the mine performs the same corrective actions as in 2017, even though the options have been proven to be ineffective. The only difference between the surface sealing now and that done in 2017 is that the thickness of the added sandy clay soil covering is now 2 m, whereas in 2017, it was 1 m. Noticeably, since the recommended thickness of clay surface sealing is 1 to 2 m, and that of sand surface sealing is 5 to 10 m (Sloss, 2015), the appropriate thickness of sandy clay surface sealing ranges from 2 to 5 m. Yet no long‐term preventive monitoring system to guarantee the successful suppression of existing fire or the prevention of future spontaneous combustion is being installed. Thus, this corrective action is unlikely to be effective.

### 3.5 Community Empowerment and Participatory Risk Management Decision

We presented the available management options and their evaluation based on US EPA's seven criteria for remedial selection to the affected villagers (Figure S21 in SI) in order for them to have information on which to base their participatory risk management decision. As shown in Table 3, they ranked grouting or injection of inert gas and inhibitor as the most preferable option for suppression of existing fire, giving the reason that this technique is an active technique to suppress the fire, while they ranked surface sealing as a secondary preferred option, since they have experienced that this technique can suppress existing fire but cannot do so for the long term, as evident from the corrective action by the mine in 2017. Interestingly, the villagers ranked excavation as the least preferable option because they do not think that it can be done practically at their site.

For proper mine waste storage and prevention of future spontaneous combustion, the villagers ranked on‐site secure landfill and off‐site disposal as the most preferable options (Table 3). Nevertheless, they recognized that these techniques are expensive and the financial constraints of the mining company may make these two options impractical. Thus, the villagers also evaluated surface sealing with long‐term preventive monitoring as a preferable option. However, they rejected the current options being performed by the mine because no long‐term preventative monitoring system is being installed to guarantee successful fire suppression. This no‐monitoring option has been proven to be unsuccessful since 2017, and the affected villagers cannot accept the same failure. The affected villagers commented that once the spontaneous combustion occurs, it takes a very long time for the mine or the regional government agency to address the problem properly. During that time, they will unavoidably be impacted by air pollution because of the proximity of their residential and agricultural land to the waste heap. Thus, a long‐term preventative monitoring system, together with an emergency response protocol to eliminate hot spots before they fully develop into areas of spontaneous combustion, is what the villagers need most. Consistent with the concern of the affected villagers, the importance of a preventive monitoring system for coal‐mine waste storage has been emphasized in multiple studies (Guo, Wen, et al., 2019; Hu & Xia, 2017; W. Lu et al., 2017; Sloss, 2015). Furthermore, several case studies in which a preventive monitoring system for coal mining and coal‐mine waste storage was implemented were proven to be successful (Lu & Qin, 2015; Ribeiro et al., 2017; Yan et al., 2020).

Taking all eight criteria into account, grouting or injection of inert gas and inhibitor for suppression of existing fire followed by off‐site disposal are ranked as the most preferable corrective actions. Nevertheless, the affected villagers can accept using surface sealing for suppression of existing fire followed by surface sealing with long‐term preventive monitoring, with the emphasis that long‐term monitoring systems must be installed and active.

## 4 Conclusion

Since 2015, spontaneous combustion from a large coal‐mine waste heap has affected the quality of life of an indigenous community in Ban Chaung, Dawei district, Myanmar. Based on historic and newly collected field and satellite data, the community ranked grouting or injection of inert gas and inhibitor for suppression of existing fire followed by off‐site disposal as the most preferable corrective actions. However, the affected villagers can accept using surface sealing with long‐term monitoring for suppression of existing fire and future spontaneous combustion, with the condition that long‐term monitoring systems must be installed and active. The mining company chose to use surface sealing for both suppression of existing fire and on‐site storage of the mine waste but did not install any long‐term monitoring system. Since this is the same correction action that was taken unsuccessfully in 2017, the affected villagers doubt whether it will be successful this time. The results of this project will be forwarded to the responsible regional Myanmar government for its evaluation of the mining company's corrective action.

This study clearly shows that a community citizen science approach provides social learning and empowers and emancipates marginalized individuals and communities. With sufficient scientific data and understanding, an empowered community can meaningfully participate in and influence a risk management decision. Their choice for risk management is rational and based on scientific information. Yet their choice is also flexible and considers the financial constraints of the polluter. Thus, to both polluters and government agencies, involving affected communities in risk management decisions by applying community citizen science is recommended for any corrective action project to gain project transparency, to elevate democratic capacity, and to obtain public trust for moving forward (Hitch et al., 2020).

## Conflict of Interest

The author declares no conflicts of interest relevant to this study.

## Supporting information

Supporting Information S1

## References

[gh2155-bib-0001] Averett, N. (2017). New blood: The promise of environmental health citizen science projects. Environmental Health Perspectives, 125(11), 112001 10.1289/EHP2484 29116929 PMC5947938

[gh2155-bib-0002] Breuer, L. , Hiery, N. , Kraft, P. , Bach, M. , Aubert, A. H. , & Frede, H.‐G. (2015). HydroCrowd: A citizen science snapshot to assess the spatial control of nitrogen solutes in surface waters. Scientific Reports, 5(1), 16503 Article. 10.1038/srep16503 26561200 PMC4642352

[gh2155-bib-0003] Cabanellas‐Reboredo, M. , Vázquez‐Luis, M. , Mourre, B. , Álvarez, E. , Deudero, S. , Amores, Á. , Addis, P. , Ballesteros, E. , Barrajón, A. , Coppa, S. , García‐March, J. R. , Giacobbe, S. , Casalduero, F. G. , Hadjioannou, L. , Jiménez‐Gutiérrez, S. V. , Katsanevakis, S. , Kersting, D. , Mačić, V. , Mavrič, B. , Patti, F. P. , Planes, S. , Prado, P. , Sánchez, J. , Tena‐Medialdea, J. , de Vaugelas, J. , Vicente, N. , Belkhamssa, F. Z. , Zupan, I. , & Hendriks, I. E. (2019). Tracking a mass mortality outbreak of pen shell *Pinna nobilis* populations: A collaborative effort of scientists and citizens. Scientific Reports, 9(1), 13355 10.1038/s41598-019-49808-4 31527825 PMC6746856

[gh2155-bib-0004] Dang, J. , Zhao, J. , Zhang, Y. , Huang, A. , Liu, X. , Zhai, X. , & Wang, C. (2016). Thermal analysis of spontaneous combustion behavior of partially oxidized coal. Process Safety and Environmental Protection, 104, 218–224. 10.1016/j.psep.2016.09.007

[gh2155-bib-0005] Deng, J. , Ma, X. , Zhang, Y. , Li, Y. , & Zhu, W. (2015). Effects of pyrite on the spontaneous combustion of coal. International Journal of Coal Science & Technology, 2(4), 306–311. 10.1007/s40789-015-0085-y

[gh2155-bib-0006] EPA‐Approved Kentucky Regulations , Chapter 50 Division for Air Quality, 82 FR 42746 7 (2017).

[gh2155-bib-0007] Fritz, S. , See, L. , Carlson, T. , Haklay, M. , Oliver, J. L. , Fraisl, D. , Mondardini, R. , Brocklehurst, M. , Shanley, L. A. , Schade, S. , Wehn, U. , Abrate, T. , Anstee, J. , Arnold, S. , Billot, M. , Campbell, J. , Espey, J. , Gold, M. , Hager, G. , He, S. , Hepburn, L. , Hsu, A. , Long, D. , Masó, J. , McCallum, I. , Muniafu, M. , Moorthy, I. , Obersteiner, M. , Parker, A. J. , Weisspflug, M. , & West, S. (2019). Citizen science and the United Nations Sustainable Development Goals. Nature Sustainability, 2(10), 922–930. 10.1038/s41893-019-0390-3

[gh2155-bib-0008] Guo, J. , Wen, H. , Zheng, X. , Liu, Y. , & Cheng, X. (2019). A method for evaluating the spontaneous combustion of coal by monitoring various gases. Process Safety and Environmental Protection, 126, 223–231. 10.1016/j.psep.2019.04.014

[gh2155-bib-0009] Guo, J. , Yan, H. , Liu, Y. , & Li, S. S. (2019). Preventing spontaneous combustion of coal from damaging ecological environment based on thermogravimetric analysis. Applied Ecology and Environmental Research, 17(4), 9051–9064.

[gh2155-bib-0010] Hitch, M. , Lytle, M. , & Tost, M. (2020). Social licence: Power imbalances and levels of consciousness—Two case studies. International Journal of Mining, Reclamation and Environment, 34(4), 238–246. 10.1080/17480930.2018.1530582

[gh2155-bib-0011] Htwe, Z. Z. (2019). Mine waste ruins 100 acres of rice fields in Sagaing. Myanmar Times.

[gh2155-bib-0012] Hu, Z. , & Xia, Q. (2017). An integrated methodology for monitoring spontaneous combustion of coal waste dumps based on surface temperature detection. Applied Thermal Engineering, 122, 27–38. 10.1016/j.applthermaleng.2017.05.019

[gh2155-bib-0013] Kong, B. , Li, Z. H. , Yang, Y. L. , Liu, Z. , & Yan, D. C. (2017). A review on the mechanism, risk evaluation, and prevention of coal spontaneous combustion in China. Environmental Science and Pollution Research International, 24(30), 23453–23470. 10.1007/s11356-017-0209-6 28924728

[gh2155-bib-0014] Leal, O. D. A. , Castilhos, R. M. V. , Pinto, L. F. S. , Pauletto, E. A. , Lemes, E. S. , & Kunde, R. J. (2016). Initial recovery of organic matter of a grass‐covered constructed soil after coal mining. Revista Brasileira de Ciência do Solo, 40, e0150384.

[gh2155-bib-0015] Lee, Y. (2019). Myanmar: Expert calls for continued pressure as situation reaches “human rights crisis”.

[gh2155-bib-0016] Li, F. , Li, X. , Hou, L. , & Shao, A. (2018). Impact of the coal mining on the spatial distribution of potentially toxic metals in farmland tillage soil. Scientific Reports, 8(1), 14,925 10.1038/s41598-018-33132-4 30297728 PMC6175947

[gh2155-bib-0017] Li, J.‐L. , Lu, W. , Cao, Y.‐J. , Kong, B. , & Zhang, Q.‐S. (2019). Method of pre‐oxidation treatment for spontaneous combustion inhibition and its application. Process Safety and Environmental Protection, 131, 169–177. 10.1016/j.psep.2019.08.013

[gh2155-bib-0018] Li, S. , Ma, X. , & Yang, C. (2019). Prediction of spontaneous combustion in the coal stockpile based on an improved metabolic grey model. Process Safety and Environmental Protection, 116, 564–577.

[gh2155-bib-0019] Lu, W. , Cao, Y.‐J. , & Tien, J. C. (2017). Method for prevention and control of spontaneous combustion of coal seam and its application in mining field. International Journal of Mining Science and Technology, 27(5), 839–846. 10.1016/j.ijmst.2017.07.018

[gh2155-bib-0020] Lu, Y. , & Qin, B. (2015). Identification and control of spontaneous combustion of coal pillars: A case study in the Qianyingzi Mine, China. Natural Hazards, 75(3), 2683–2697. 10.1007/s11069-014-1455-2

[gh2155-bib-0021] Lui, C. , Li, S. , Qiao, Q. , Wang, J. , & Pan, Z. (1998). Management of spontaneous combustion in coal mine waste tips in China. Water, Air, and Soil Pollution, 103(1–4), 441–444.

[gh2155-bib-0022] Melody, S. M. , & Johnston, F. H. (2015). Coal mine fires and human health: What do we know? International Journal of Coal Geology, 152, 1–14. 10.1016/j.coal.2015.11.001

[gh2155-bib-0023] Meyer, C. (2018). Overview of TVOC and indoor air quality. Retrieved from San Jose, CA:

[gh2155-bib-0024] Ministry of Natural Resources and Environmental Conservation . (2015). National Environmental Quality (Emission) Guidelines. Retrieved from

[gh2155-bib-0025] Nang, S. , & Paddock, R. C. (2019). Myanmar Jade Mine Disaster Feared to Have Killed More Than 50. The New York Times.

[gh2155-bib-0026] National Advisory Council for Environmental Policy and Technology . (2016). Environmental protection belongs to the public: A vision for citizen science at EPA. Retrieved from Washington DC:

[gh2155-bib-0027] Nguyen, T. T. , & Vu, T. D. (2019). Use of hot spot analysis to detect underground coal fires from Landsat‐8 TIRS data: A case study in the Khanh Hoa coal field, North‐East of Vietnam. Environment and Natural Resources Journal, 17(3), 1–10. 10.32526/ennrj.17.3.2019.17

[gh2155-bib-0028] Núñez‐Gómez, D. , Lapolli, F. R. , Nagel‐Hassemer, M. E. , & Lobo‐Recio, M. A. (2020). Optimization of Fe and Mn removal from coal acid mine drainage (AMD) with waste biomaterials: Statistical modeling and kinetic study. Waste and Biomass Valorization, 11(3), 1143–1157. 10.1007/s12649-018-0405-8

[gh2155-bib-0029] Onifade, M. , & Genc, B. (2019). A review of spontaneous combustion studies—South African context. International Journal of Mining, Reclamation and Environment, 33(8), 527–547. 10.1080/17480930.2018.1466402

[gh2155-bib-0030] Otwong, A. , & Phenrat, T. (2017). Comparative analysis of public participation in the EIA process for Thai overseas investment projects: Krabi coal terminal, Hongsa coal power plant, and Dawei special economic zone. Impact Assessment and Project Appraisal, 35(4), 325–339. 10.1080/14615517.2017.1354641

[gh2155-bib-0031] Pone, J. D. N. , Hein, K. A. A. , Stracher, G. B. , Annegarn, H. J. , Finkleman, R. B. , Blake, D. R. , McCormack, J. K. , & Schroeder, P. (2007). The spontaneous combustion of coal and its by‐products in the Witbank and Sasolburg coalfields of South Africa. International Journal of Coal Geology, 72(2), 124–140. 10.1016/j.coal.2007.01.001

[gh2155-bib-0032] Qin, B. , Li, L. , Ma, D. , Lu, Y. , Zhong, X. , & Jia, Y. (2016). Control technology for the avoidance of the simultaneous occurrence of a methane explosion and spontaneous coal combustion in a coal mine: A case study. Process Safety and Environmental Protection, 103, 203–211. 10.1016/j.psep.2016.07.005

[gh2155-bib-0033] Querol, X. , Zhuang, X. , Font, O. , Izquierdo, M. , Alastuey, A. , Castro, I. , van Drooge, B. L. , Moreno, T. , Grimalt, J. O. , Elvira, J. , Cabañas, M. , Bartroli, R. , Hower, J. C. , Ayora, C. , Plana, F. , & López‐Soler, A. (2011). Influence of soil cover on reducing the environmental impact of spontaneous coal combustion in coal waste gobs: A review and new experimental data. International Journal of Coal Geology, 85(1), 2–22. 10.1016/j.coal.2010.09.002

[gh2155-bib-0034] Ribeiro, J. , Ferreirada Silva, E. , & Flores, D. (2010). Burning of coal waste piles from Douro Coalfield (Portugal): Petrological, geochemical and mineralogical characterization. International Journal of Coal Geology, 81(4), 359–372. 10.1016/j.coal.2009.10.005

[gh2155-bib-0035] Ribeiro, J. , Viveiros, D. , Ferreira, J. , Lopez‐Gil, A. , Dominguez‐Lopez, A. , Martins, H. F. , Perez‐Herrera, R. , Lopez‐Aldaba, A. , Duarte, L. , Pinto, A. , Martin‐Lopez, S. , Baierl, H. , Jamier, R. , Rougier, S. , Auguste, J.‐L. , Teodoro, A. , Gonçalves, J. , Esteban, O. , Santos, J. , Roy, P. , Lopez‐Amo, M. , Gonzalez‐Herraez, M. , Baptista, J. , & Flores, D. (2017). ECOAL project—Delivering solutions for integrated monitoring of coal‐related fires supported on optical fiber sensing technology. Applied Sciences, 7(9), 956 10.3390/app7090956

[gh2155-bib-0036] Sahoo, P. K. , Equeenuddin, S. M. , & Powell, M. A. (2016). Trace elements in soils around coal mines: Current scenario, impact and available techniques for management. Current Pollution Reports, 2(1), 1–14. 10.1007/s40726-016-0025-5

[gh2155-bib-0037] Saraf, A. K. , Prakash, A. , Sengupta, S. , & Gupta, R. P. (1995). Landsat‐TM data for estimating ground temperature and depth of subsurface coal fire in the Jharia coalfield, India. International Journal of Remote Sensing, 16(12), 2111–2124. 10.1080/01431169508954545

[gh2155-bib-0038] Sasaki, K. , & Sugai, Y. (2011). Equivalent oxidation exposure—Time for low temperature spontaneous combustion of coal In Ahsan A. (Ed.), Heat analysis and thermodynamic effects, (pp. 235–254). London: IntechOpen 10.5772/20308

[gh2155-bib-0039] Sloss, L. L. (2015). Assessing and managing spontaneous combustion of coal. Retrieved from London:

[gh2155-bib-0040] Song, Z. , & Kuenzer, C. (2014). Coal fires in China over the last decade: A comprehensive review. International Journal of Coal Geology, 133, 72–99. 10.1016/j.coal.2014.09.004

[gh2155-bib-0041] Staples, G. W. , & Bevacqua, R. F. (2006). Areca catechu (betel nut palm) In Elevitch C. R. (Ed.), Traditional trees of Pacific Islands: Their culture, environment, and use, (pp. 1–17). Honolulu, HI: Permanent Agriculture Resources.

[gh2155-bib-0042] Stracher, G. B. , Prakash, A. , & Sokol, E. V. (2010). Coal and peat fires: A global perspective: Volume 1: Coal—Geology and combustion. Amsterdam: Elsevier Science.

[gh2155-bib-0043] Stracher, G. B. , Prakash, A. , & Sokol, E. V. (2015). Coal and peat fires: A global perspective: Volume 3: Case studies—Coal fires. Amsterdam: Elsevier Science.

[gh2155-bib-0044] United States Environmental Protection Agency (1990). A guide to selecting superfund remedial actions. Retrieved from Washington DC:

[gh2155-bib-8845] US EPA (2017). EPA–Approved Kentucky Regulations, Chapter 50 Division for Air Quality, 401 KAR 53:005, 82 FR 42746. Retrieved from https://www.epa.gov/sites/production/files/2017-12/documents/chapter_53-2017.pdf

[gh2155-bib-0045] US EPA . (2018). Superfund. Retrieved from https://www.epa.gov/superfund

[gh2155-bib-0046] Wen, H. , Guo, J. , Jin, Y. , Wang, K. , Zhang, Y. , & Zheng, X. (2017). Experimental study on the influence of different oxygen concentrations on coal spontaneous combustion characteristic parameters. International Journal of Oil, Gas and Coal Technology, 16(2), 187–202. 10.1504/IJOGCT.2017.086320

[gh2155-bib-0047] Xi, Z. , & Li, A. (2016). Characteristics of thermoplastic powder in an aqueous foam carrier for inhibiting spontaneous coal combustion. Process Safety and Environmental Protection, 104, 268–276. 10.1016/j.psep.2016.09.012

[gh2155-bib-0048] Yan, S. , Shi, K. , Li, Y. , Liu, J. , & Zhao, H. (2020). Integration of satellite remote sensing data in underground coal fire detection: A case study of the Fukang region, Xinjiang, China. Frontiers in Earth Science, 14, 1–12.

[gh2155-bib-0049] Yang, F. , Lai, Y. , & Song, Y. (2019). Determination of the influence of pyrite on coal spontaneous combustion by thermodynamics analysis. Process Safety and Environmental Protection, 129, 163–167. 10.1016/j.psep.2019.06.023

[gh2155-bib-0050] Zhai, X. , Wu, S. , Wang, K. , Drebenstedt, C. , & Zhao, J. (2017). Environment influences and extinguish technology of spontaneous combustion of coal gangue heap of Baijigou coal mine in China. Energy Procedia, 136, 66–72. 10.1016/j.egypro.2017.10.326

[gh2155-bib-0051] Zhang, X. (2013). Gaseous emissions from coal stockpiles, IEA Clean Coal Centre (29 pages). Retrieved from https://www.iea-coal.org/report/gaseous-emissions-from-coal-stockpiles-ccc-213/

[gh2155-bib-0052] Zhu, H. , Sheng, K. , Zhang, Y. , Fang, S. , & Wu, Y. (2018). The stage analysis and countermeasures of coal spontaneous combustion based on “five stages” division. PLoS ONE, 13(8), e0202724 10.1371/journal.pone.0202724 30138357 PMC6107200

