# Supplementary material for: Community Citizen Science for Risk Management of a Spontaneously Combusting Coal‐Mine Waste Heap in Ban Chaung, Dawei District, Myanmar
Source: Geohealth. 2020 Jun 1;4(6):e2020GH000249. doi: 10.1029/2020GH000249 (PMC7291502; doi:10.1029/2020GH000249)
Supplement: Supplementary file 1 — Supporting Information S1 [file GH2-4-e2020GH000249-s001.docx]

**Supporting Information for**

**Community Citizen Science for Risk Management of Spontaneously Combusting Coal-Mine Waste Heap in Ban Chaung, Dawei District, Myanmar**

*Tanapon Phenrat^1,2^*^*^

^1^Research Unit for Integrated Natural Resources Remediation and Reclamation (IN3R), Department of Civil Engineering, Faculty of Engineering, Naresuan University, Phitsanulok, Thailand, 65000

^2^Center of Excellence for Sustainability of Health, Environment and Industry (SHEI), Faculty of Engineering, Naresuan University, Phitsanulok, Thailand, 65000

***GeoHealth***

***Special Collection: Mining and Planetary Health***

***Corresponding author: Tanapon Phenrat (**[**pomphenrat@gmail.com**](mailto:pomphenrat@gmail.com)**)**

**(6655)964057(ph) (6655)964002(fax)**

**
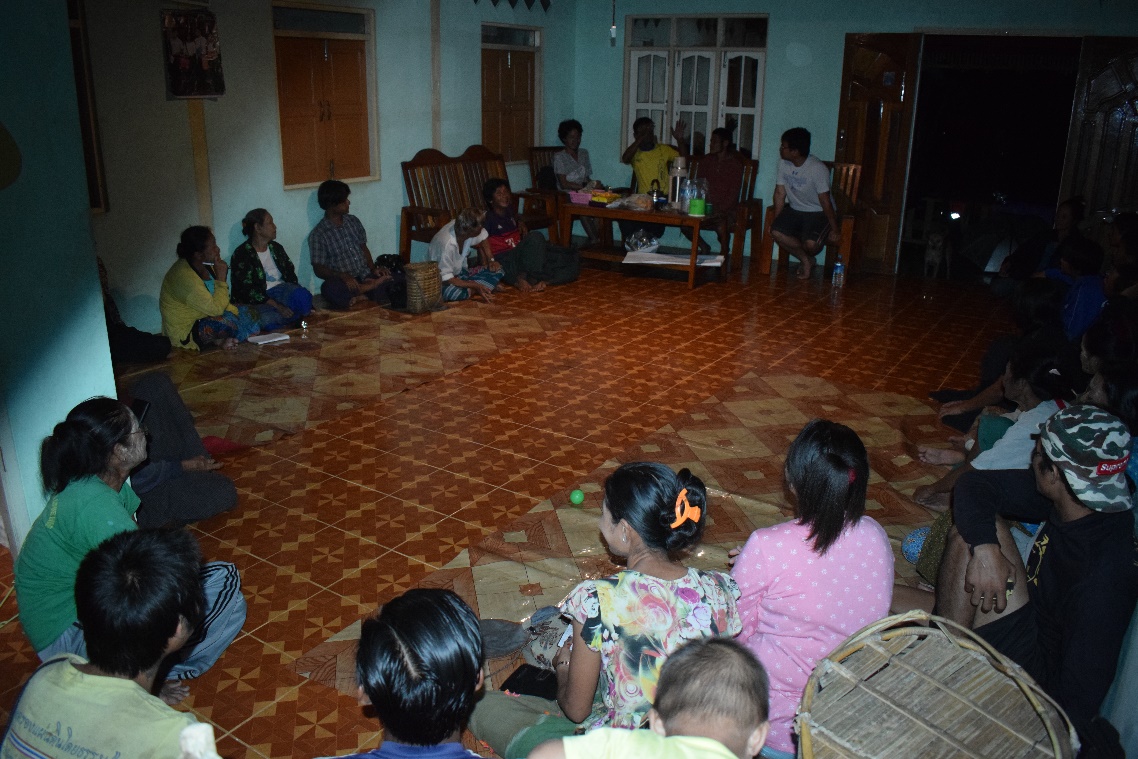
**

**
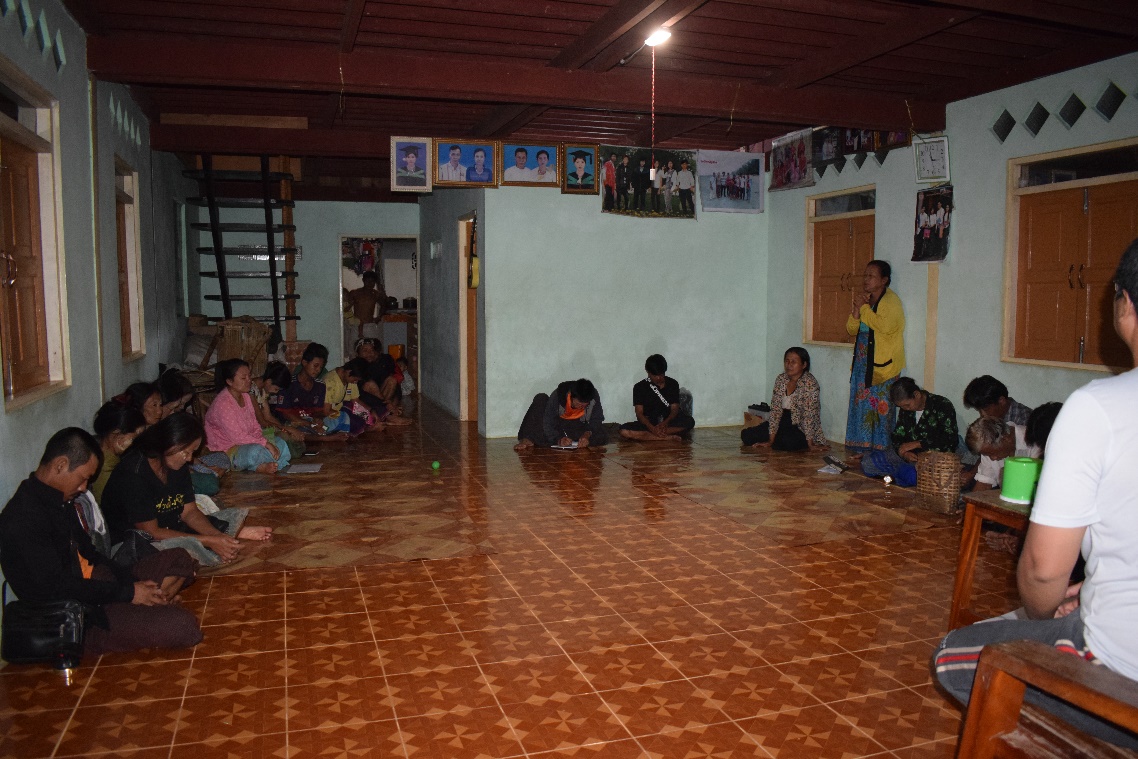
**

**Figure S1 Meeting with representatives of the affected community, some of which are members of the local monitoring group, to establish the objectives of the community citizen science project**

**
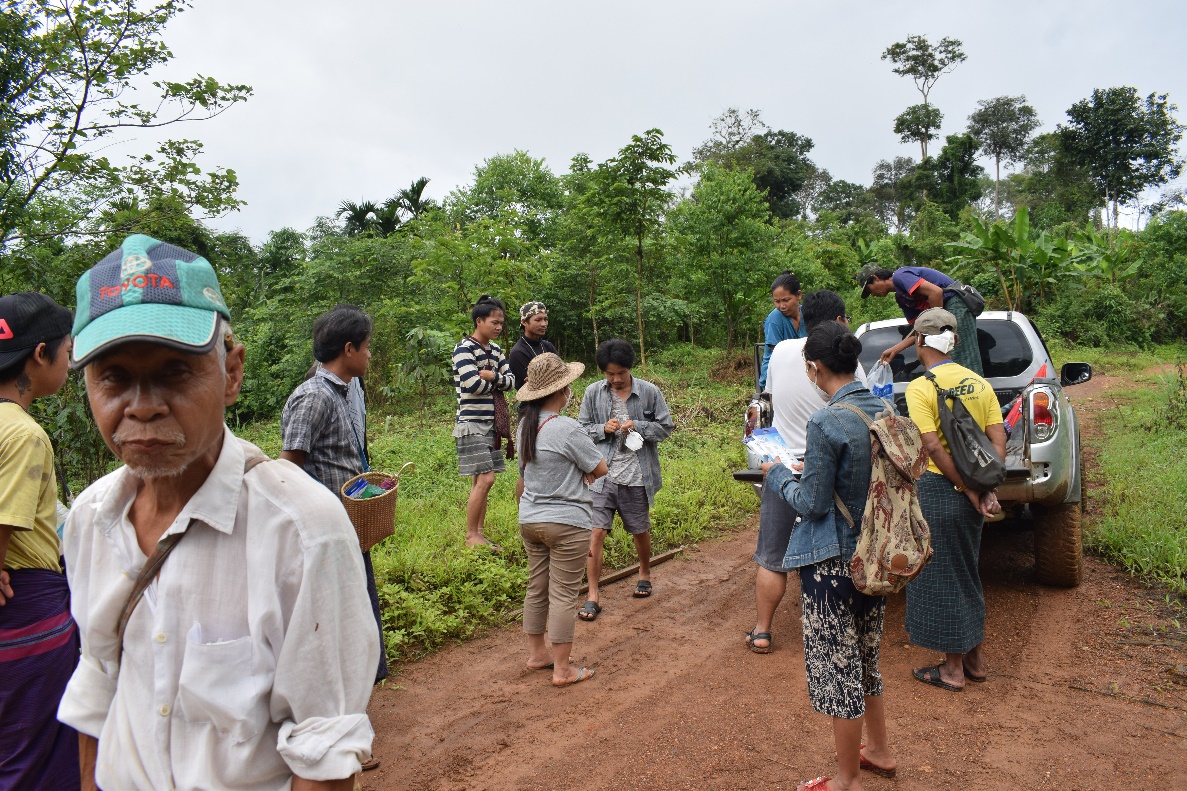
**

**
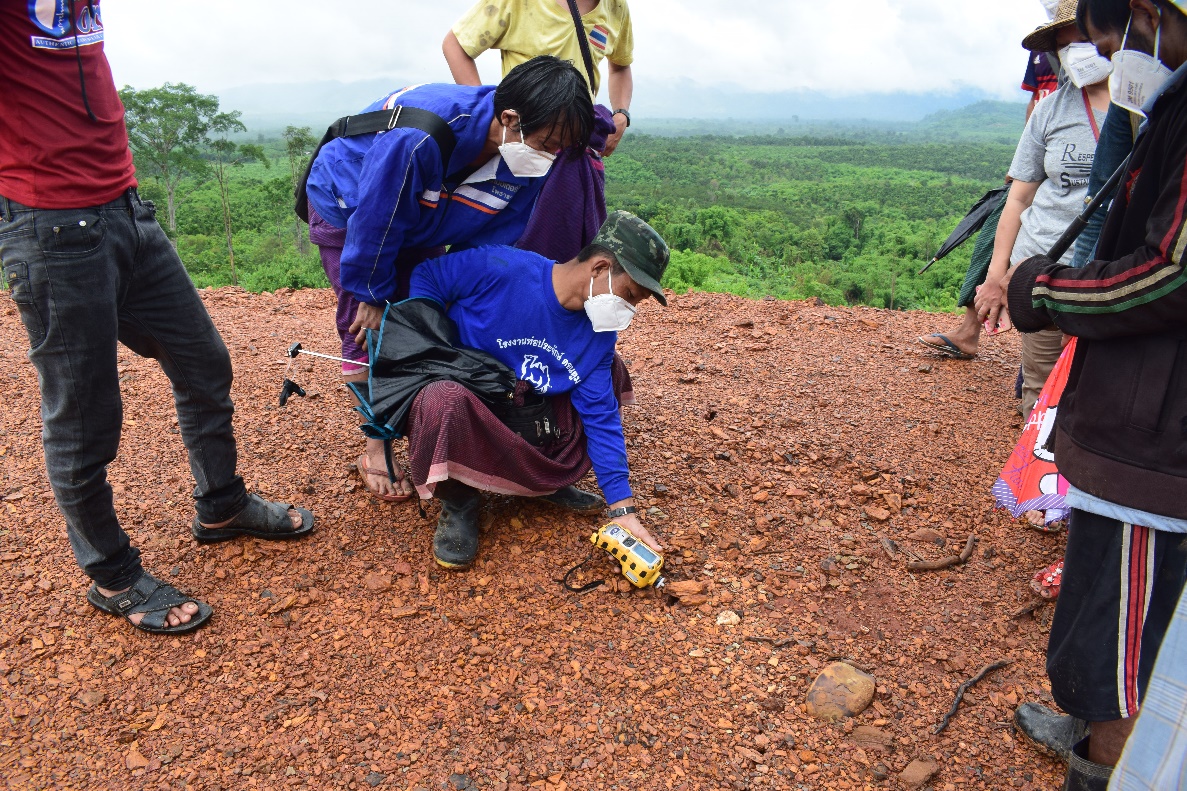
**

**Figure S2 The local monitoring group on the field PA/SI in 2019**

**Table S1 Sampling locations**

| **No.** | **Description** | **Location** | **GPS Position (UTM)** |
| --- | --- | --- | --- |
| 1 | Control | Control area | 47P 458659 1538609 |
| 2 | Part of waste heap with insufficient soil cover | At the base of the waste heap | 47P 466633 1529347 |
| 3 | A crack | on top of the waste heap | 47P 466515 1529416 |
| 4 | A crack | on top of the waste heap | 47P 466512 1529412 |
| 5 | A small waste pile in open space | In the middle layer of the waste heap | 47P 466536 1529473 |
| 6 | Open space | on top of the waste heap | 47P 466465 1529434 |
| 7 | Mine lake | Close to the coal mine | 47P 466301 1529321 |
| 8 | Open space | Affected agricultural area, 0.5 km from the waste heap | 47P 466972 1529289 |
| 9 | A residential area | 0.5 km from the waste heap | 47P 466547 1528947 |
| 10 | Playground | a school 1.2 km from the waste heap | 47P 466839 1528234 |
| 11 | A residential area | 1.3 km from the waste heap | 47P 466322 1528164 |
| 12 | Highly affected surface water | Drainage from coal mining and waste storage area | 47P 466072 1529251 |
| 13 | Moderately affected surface water | Natural creek at the back of the mining and waste storage area | 47P 465853 1529375 |

**
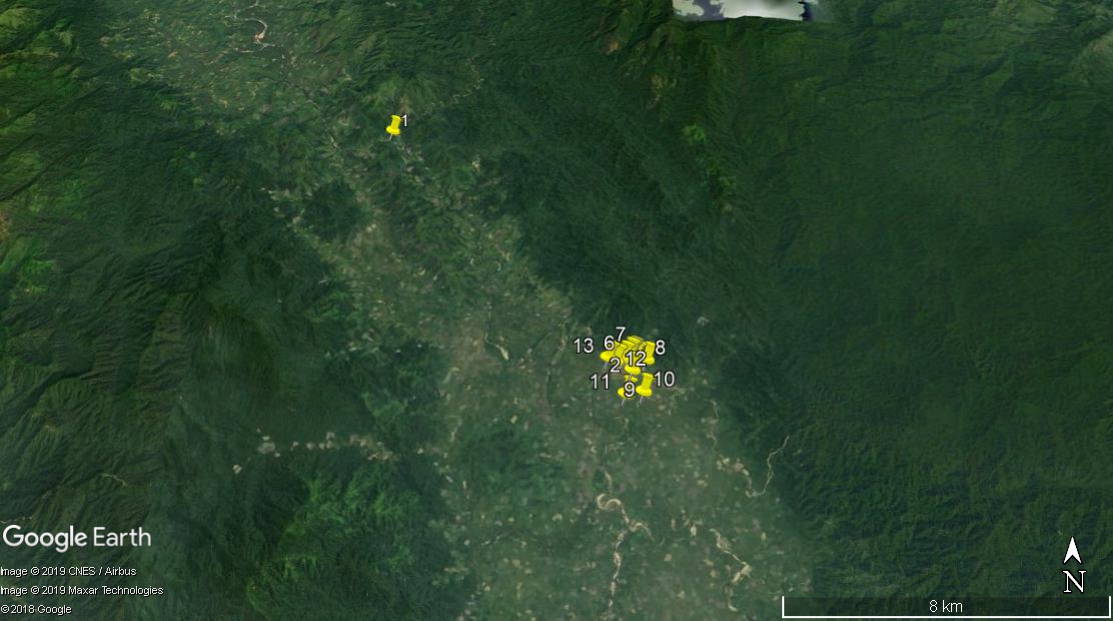
**

**
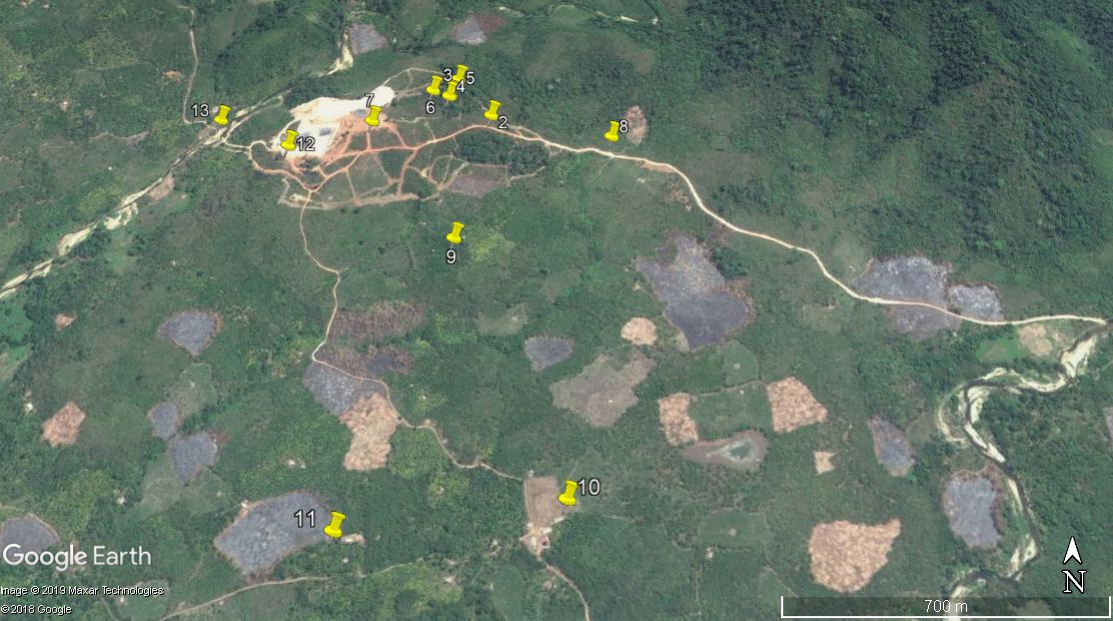
**

**Figure S3 Sampling locations (see also Table S1)**

**
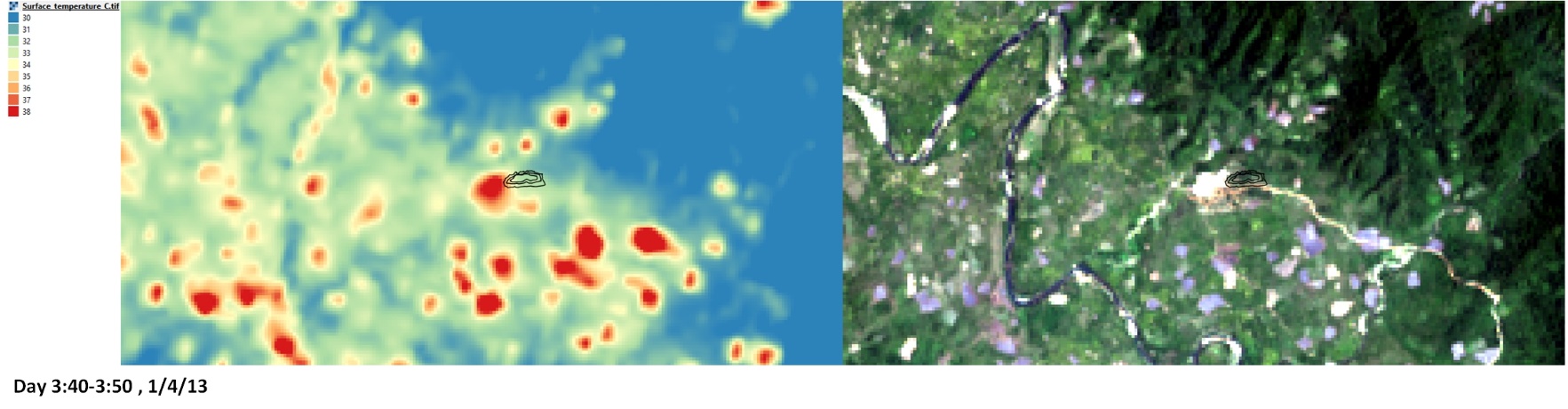
**

**(a)**

**(b)**

**
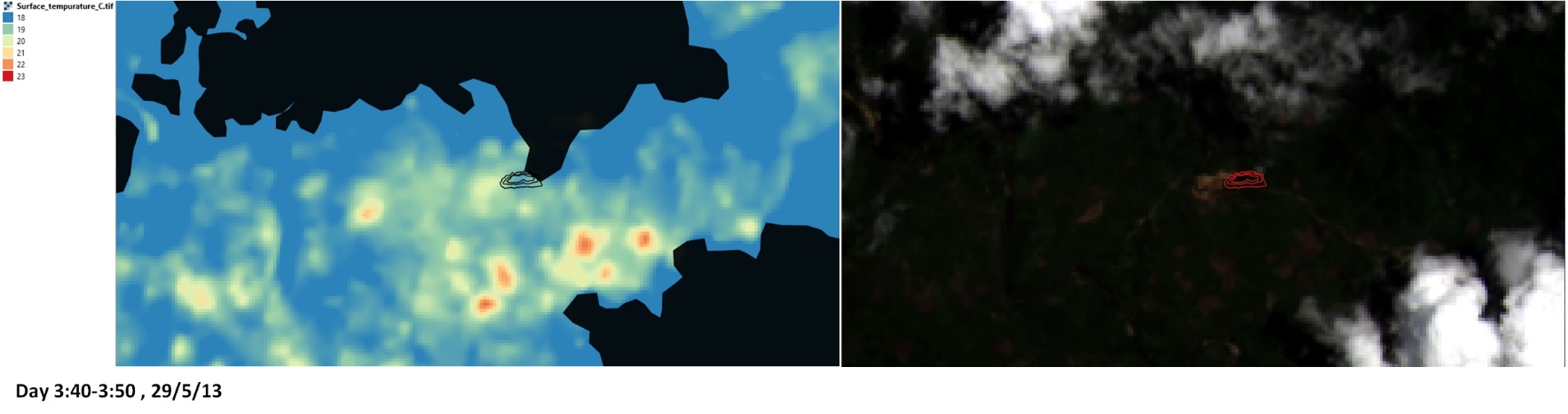
**

**(c)**

**
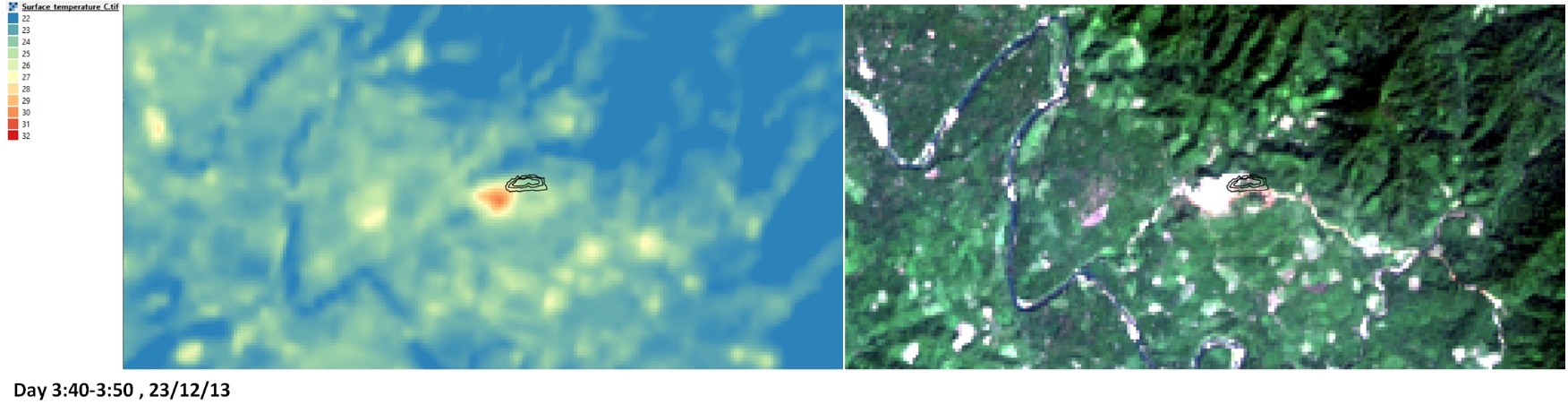
**

**Figure S4 Surface temperatures according to Landsat-8 TIRS in 2013 for (a) summer, (b) monsoon, and (c) winter. The polygon represents the location of the mine waste heap, although no waste heap existed in 2013.**

**
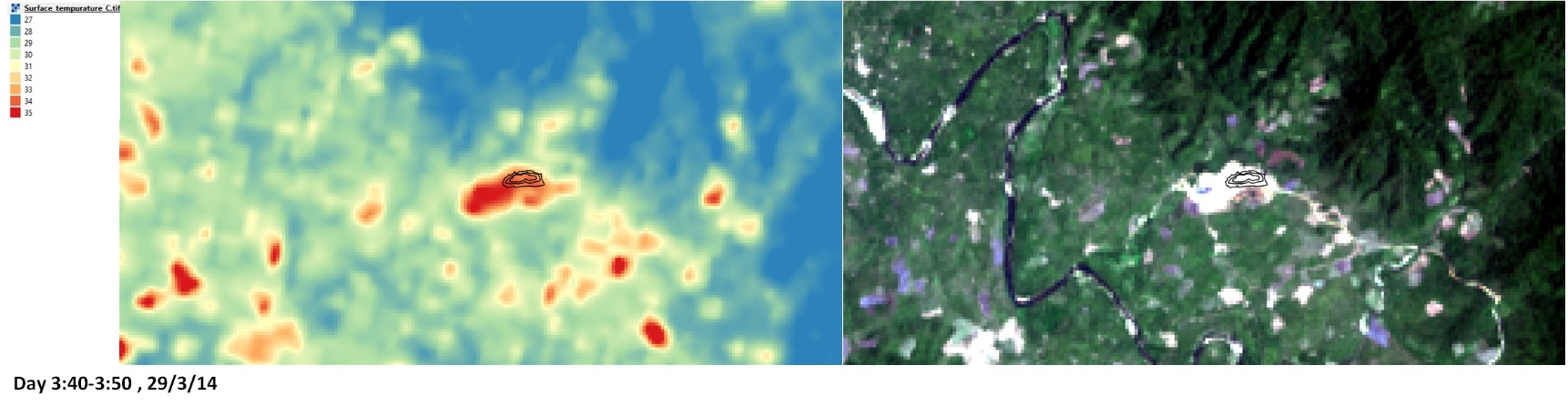
**

**(a)**

**(b)**

**
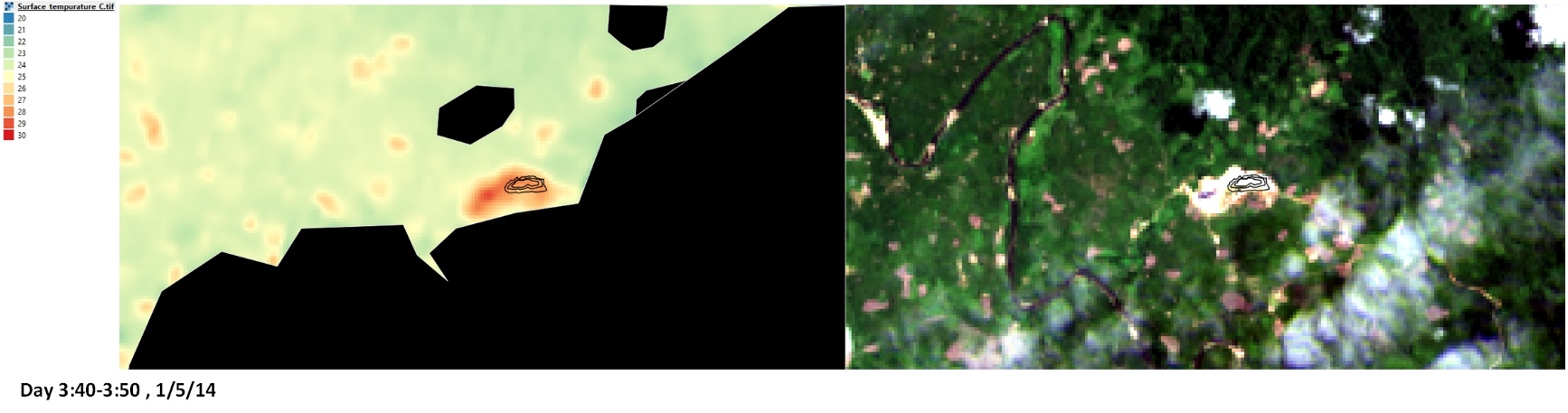
**

**
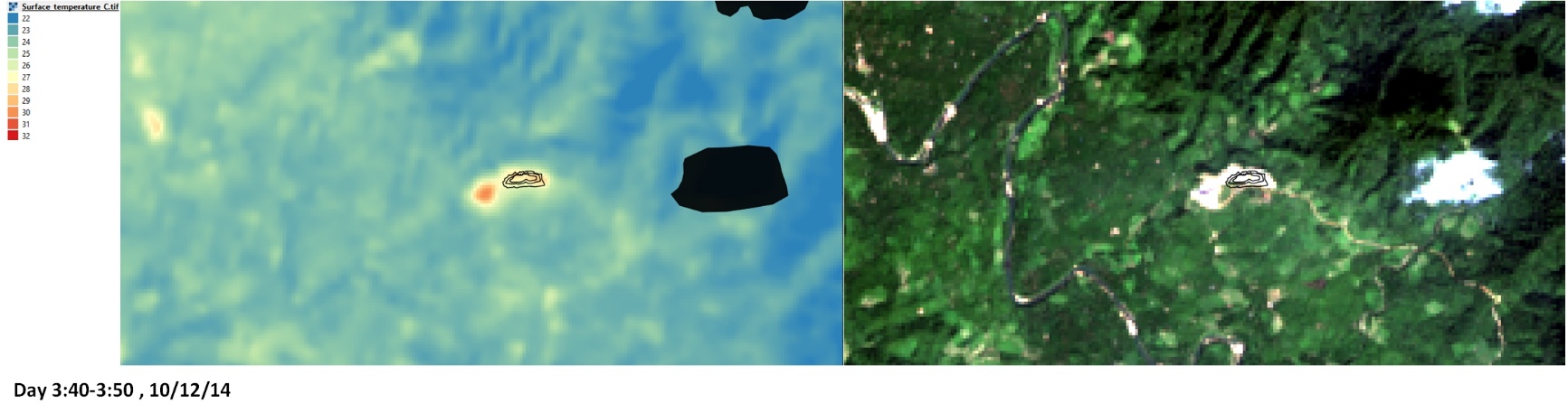
**

**(c)**

**Figure 5 Surface temperatures according to Landsat-8 TIRS in 2014 for (a) summer, (b) monsoon, and (c) winter. The polygon represents the location of the mine waste heap.**

**
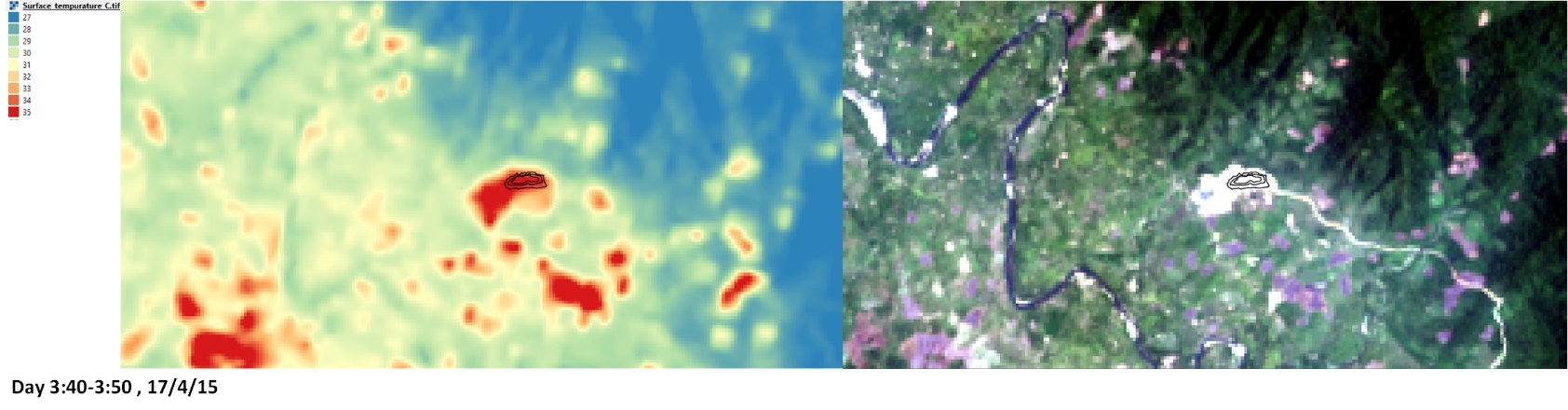
**

**(a)**

**(b)**

**
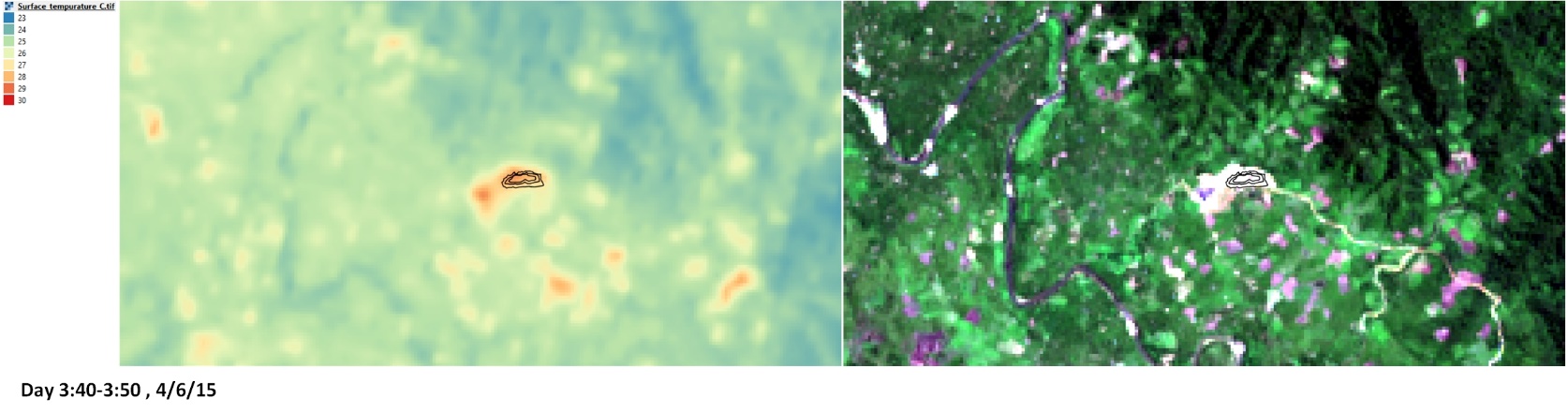
**

**(c)**

**
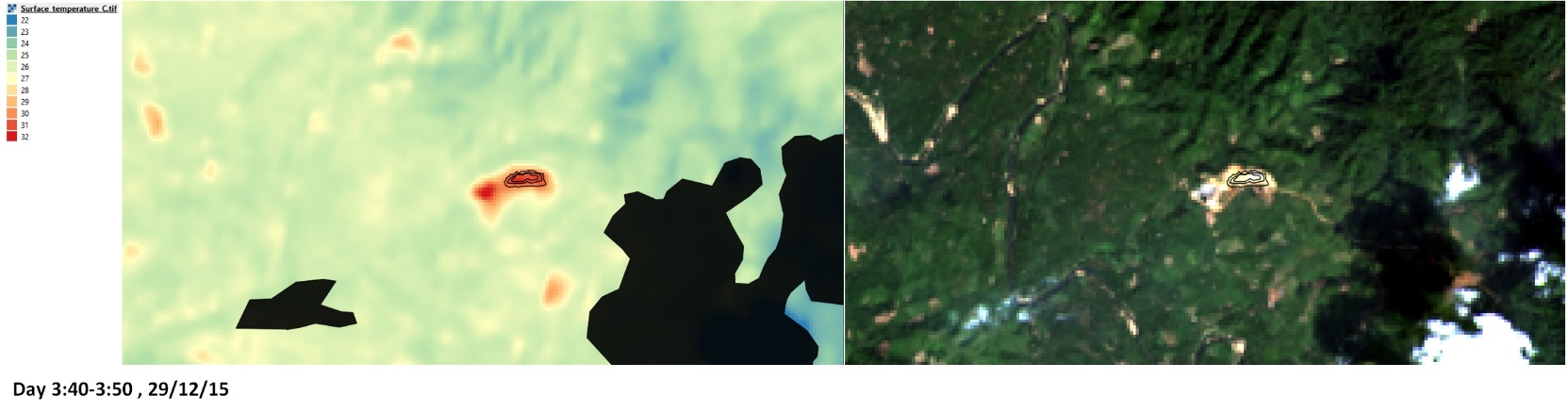
**

**Figure S6 Surface temperatures according to Landsat-8 TIRS in 2015 for (a) summer, (b) monsoon, and (c) winter. The polygon represents the location of the mine waste heap.**

**
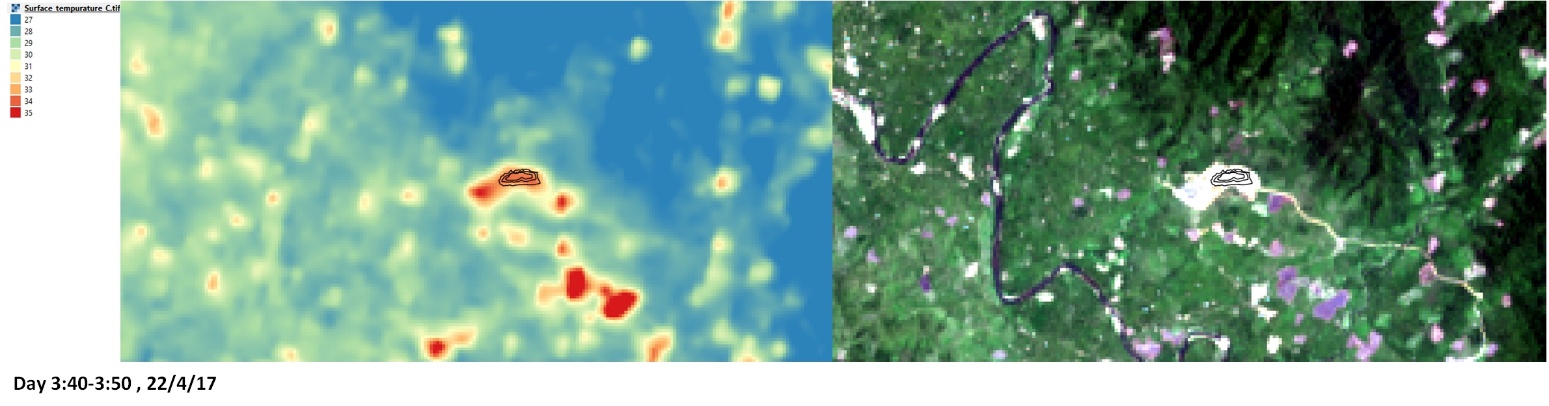
**

**(a)**

**(b)**

**
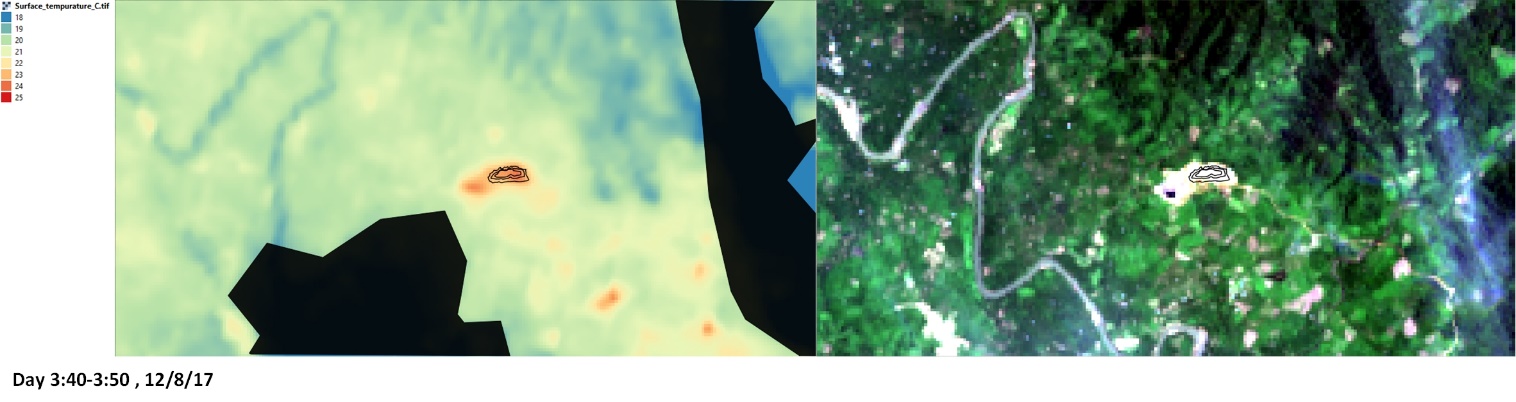
**

**
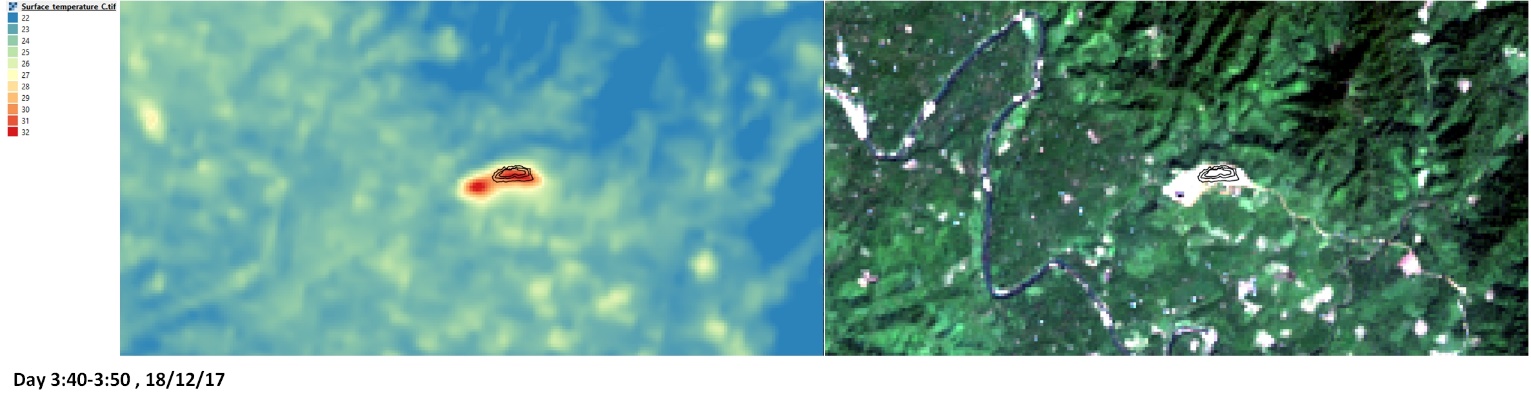
**

**(c)**

**Figure S7 Surface temperatures according to Landsat-8 TIRS in 2017 for (a) summer, (b) monsoon, and (c) winter. The polygon represents the location of the mine waste heap.**

**
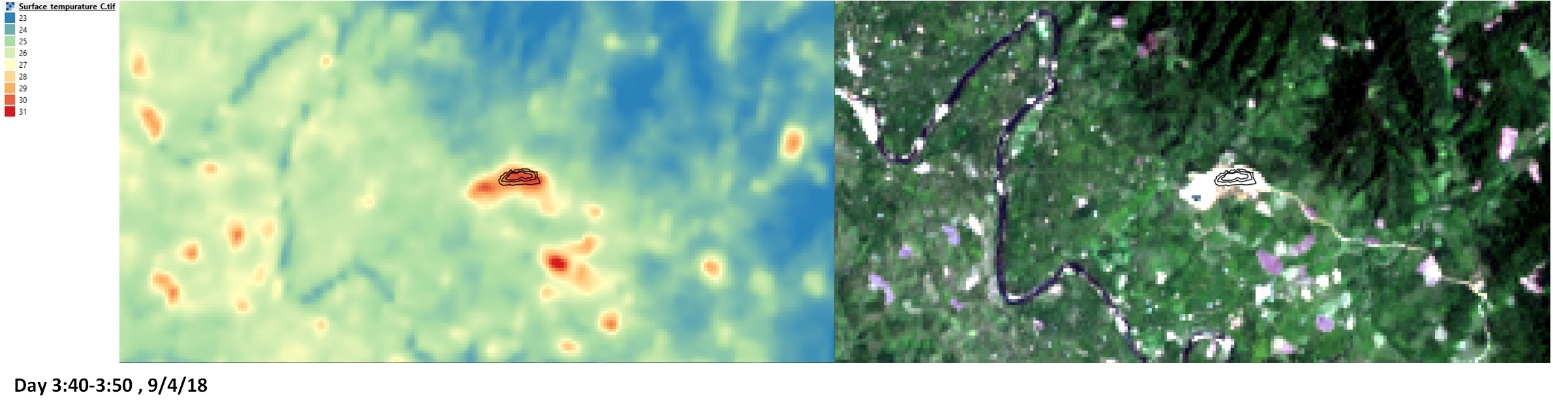
**

**(a)**

**(b)**

**(c)**

**
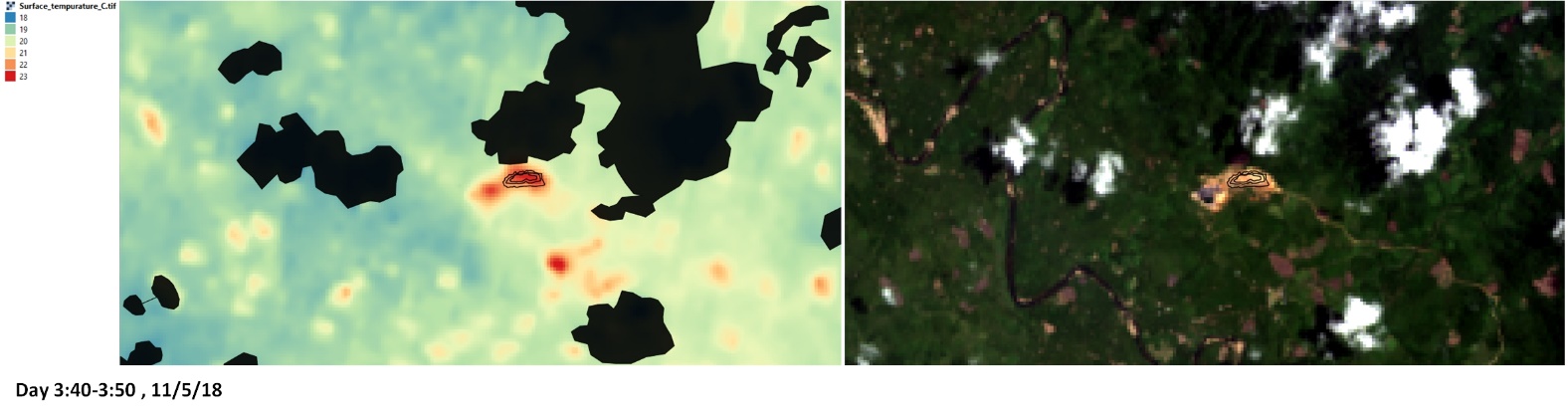
**

**
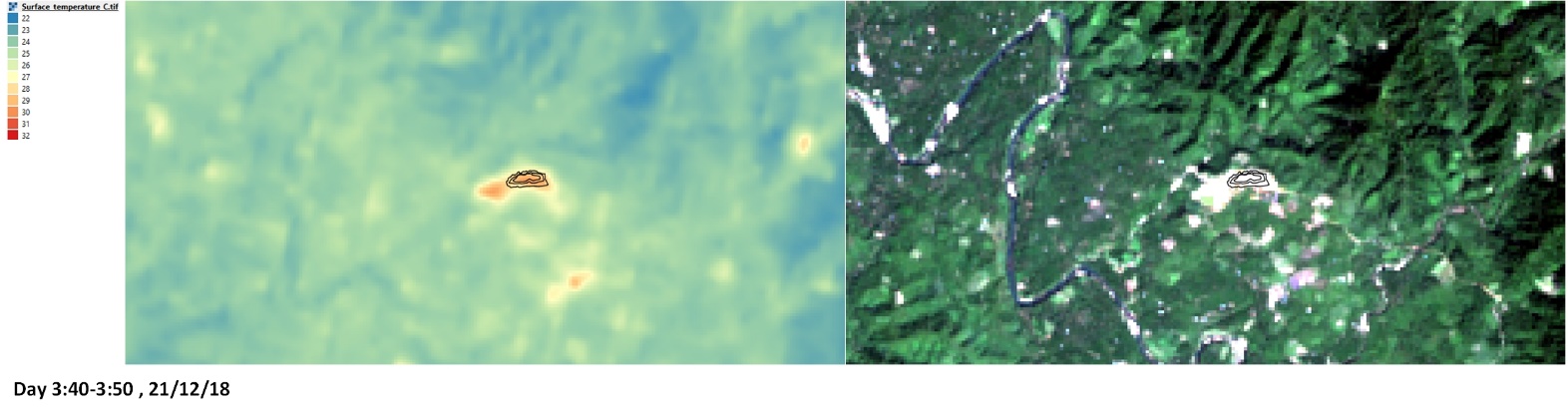
**

**Figure S8 Surface temperatures according to Landsat-8 TIRS in 2018 for (a) summer, (b) monsoon, and (c) winter. The polygon represents the location of the mine waste heap.**

**(a)**

**(b)**

**
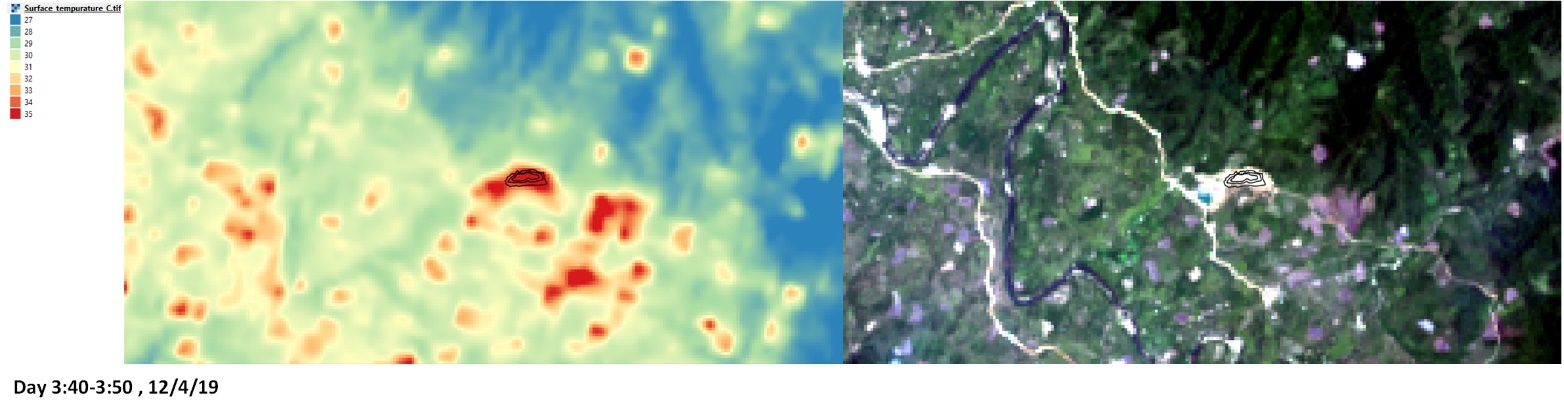
**

**
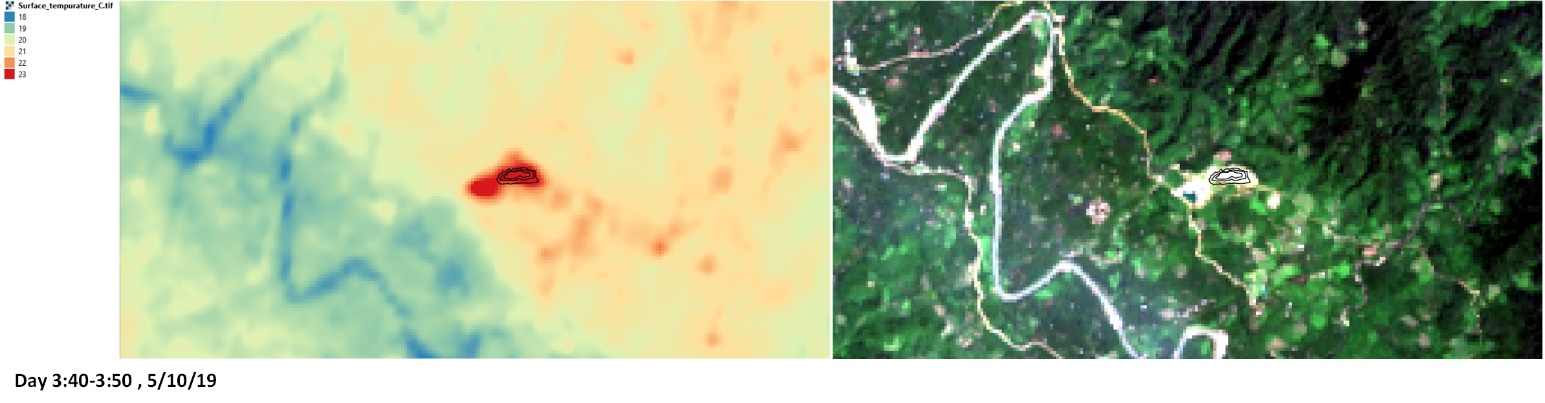
**

**Figure S9 Surface temperatures according to Landsat-8 TIRS in 2019 for (a) summer and (b) monsoon. The polygon represents the location of the mine waste heap.**

**Table S2 Scoring System for Remedial Options based on USEPA’s Nine Criteria**

| **Criteria** | **Scoring System** | **Note** |
| --- | --- | --- |
| Overall protection of human health and the environment | No substantial release of toxic air or water pollutants during the existing fire suppression or mine waste storage (score = 5)  Substantial release of toxic air or water pollutants during the existing fire suppression or mine waste storage. However, with appropriate management, the release is still acceptable, i.e., unlikely to pose health and environmental threat (score = 3)  Substantial release of toxic air or water pollutants during the existing fire suppression or mine waste storage. Since management is challenging, the release may post unacceptable risk from time to time, i.e., likely to pose health and environmental threat occasionally (score = 1)  Unacceptable release of toxic air or water pollutants during the existing fire suppression activity or mine waste storage, i.e., likely to pose health and environmental threat continuously (score = 0) | A selected candidate must have a score > 0 |
| Compliance with applicable or relevant and appropriate requirements | The emission of some toxic air or water pollutants is unlikely beyond the regulated value by law for most of the time (during the existing fire suppression activity or mine waste storage) (score = 5)  The emission of some toxic air or water pollutants may be beyond the regulated value by law for certain, short periods (during the existing fire suppression activity or mine waste storage) (score = 3)  There is a risk that the emission of some toxic air or water pollutants may be beyond the regulated value by law for most of the time (during the existing fire suppression activity or mine waste storage). However, the risk is still uncertain and requires further study (score = 1)  The emission of some toxic air or water pollutants is beyond the regulated value by law for most of the time (during the existing fire suppression activity or mine waste storage) (score = 0) | A selected candidate must have a score > 0 |
| Long-term effectiveness and permanence | For existing fire suppression—  The suppression technique appears to have a substantial residual effect on decreasing the chance of future spontaneous combustion (Score = 5)  The suppression technique appears to have some residual effect on decreasing the chance of future spontaneous combustion (Score = 3)  The suppression technique has almost no impact on decreasing the chance of future spontaneous combustion (Score = 0)  For mine waste storage:  Complete removal of long-term potential of spontaneous combustion and water contamination (Score = 5)  Reduce major chance of long-term potential of future spontaneous combustion and water contamination with a reliable monitoring system and emergency response (Score = 4)  Potential for future spontaneous combustion and water contamination remains, but appropriate monitoring and emergency response is also effective (Score = 3)  Potential for future spontaneous combustion and water contamination remains without appropriate monitoring and emergency response (Score = 0) |  |
| Reduction of toxicity, mobility, or volume through treatment | For existing fire suppression:  Completely inhibit or eliminate the source of spontaneous combustion (Score = 5)  Majorly inhibit or eliminate the source of spontaneous combustion (Score = 3)  No impact on the amount or reactivity of the source of spontaneous combustion (Score = 0)  For mine waste storage:  Complete removal of mine waste from the area (i.e., off-site management) (Score = 5)  On-site management with treatment of coal to reduce spontaneous combustion (Score = 3)  On-site management without treatment of coal to reduce spontaneous combustion (Score = 0) |  |
| Short-term effectiveness | For existing fire suppression:  The suppression technique has an immediate impact on extinguishing spontaneous combustion (Score = 5)  The suppression technique has a gradual impact on extinguishing spontaneous combustion (days to weeks) (Score = 3)  The suppression technique has a relatively slow impact on extinguishing spontaneous combustion (weeks to months) (Score = 0)  For mine waste storage:  Immediately provide proper storage conditions for mine waste (Score = 5)  Take moderate time to establish proper storage conditions for mine waste (Score = 3)  Take a relatively long time to establish proper storage conditions for mine waste (Score = 0) |  |
| Implement ability | Very easy to implement; not technically complicated; not dangerous; has been conducted in several cases; very reliable technique (Score = 5)  Moderately difficult to implement; some technical challenges; some risk during implementation; has been conducted in some cases; moderately reliable technique (Score = 3)  Difficult to implement; several technical challenges; involves significant risk during implementation; only pilot cases have been done; not very reliable technique (Score = 0) |  |
| Cost | For existing fire suppression:  Estimated cost <0.5 million USD (Score = 5)  0.5≤Estimated cost <1.5 million USD (Score = 4)  1.5≤Estimated cost <2.5 million USD (Score = 3)  2.5≤Estimated cost <3.5 million USD (Score = 2)  3.5≤Estimated cost <4.5 million USD (Score = 1)  Estimated cost ≥4.5 million USD (Score = 0)  For mine waste storage:  Estimated cost <2.5 million USD (Score = 5)  2.5≤Estimated cost <7.5 million USD (Score = 4)  7.5≤Estimated cost <15 million USD (Score = 3)  15≤Estimated cost <30 million USD (Score = 2)  30≤Estimated cost <60 million USD (Score = 1)  Estimated cost ≥60 million USD (Score = 0) |  |

**Options for Existing Fire Suppression**

There are four options for existing fire suppression to be evaluated in this study. Yet, for all except the first option (no action), the first step is conducting preparation to guarantee fire-fighting work is conducted safely. The preparation steps include surface cooling by pouring water on the waste heap and blasting and removing unstable overlying rocks, if any, above the working area (Song and Kuenzer, 2014). In addition, the preparation of qualified and experienced fire-fighting crews equipped with the appropriate level of protective gear is essential. Only qualified crews with appropriate training should be allowed to work on this dangerous mission. It is also crucial to communicate the safety plan with the nearby villagers to know the field action and safety protocol of fire suppression.

1. **No action**

No action is an option that needs to be assessed according to USEPA remedial alternative consideration (United States Environmental Protection Agency, 1990). For this mine waste heap, if no action is performed, spontaneous combustion will occur, causing the health and environmental impacts discussed previously. This is unacceptable for the first two evaluation criteria and cannot be an appropriate remedial option (United States Environmental Protection Agency, 1990).

1. **Excavation**

After the preparation step, excavation can be employed to extinguish smoldering and spontaneous combustion. This method is the most effective for extinguishing fires, especially during early combustion. It involves digging deep into the burning layers of the coal-mine waste heap, spreading the coal-mine waste on a flat (pre-prepared) area with proper ventilation, and extinguishing the fire with a high-pressure water spray (see Figure S10). For this approach, a flat, prepared plot of land and heavy machines, including a backhoe, bulldozer, excavator dump truck, and water truck with a high-pressure water spray, are necessary. Although this method is straightforward, it is costly, environmentally disruptive, and dangerous. Working in close proximity to superheated burning coal waste poses immediate safety issues for personnel and equipment. In addition, work operations can easily cause the fire to escalate or result in forest or range fires. Furthermore, it may increase the release of toxic gaseous pollutants and particulate matter that were once contained in the waste heap. Thus, a health and safety plan for both workers and nearby villagers is critical for this method. Protective gear (especially for inhalation protection) are needed for both workers and nearby villagers. Thus, the water truck must be ready for emergency response while planning on excavation steps (for example, excavation from top to bottom or left to right) is critical. This method will work for the coal waste heap in this study since there is a large and easily accessible water source near the site. Nevertheless, careful consideration is needed for choosing the prepared area for fire extinguishment as it must be far away from the forest to avoid forest fires. This method is efficient if the mine operators want to move the coal waste off-site or to build a new landfill for coal storage on site.

Evaluation based on seven USEPA criteria

Based on the literature and the environmental condition of the study site, we assigned the following scores to this technique.

**Table S3 Evaluation of excavation based on seven USEPA criteria**

| **Criteria** | **Score Given** | **Rational** | **Note** |
| --- | --- | --- | --- |
| Overall protection of human health and the environment | 3 | Some toxic air pollutants are likely to be emitted, but with proper management, the risk should be acceptable. |  |
| Compliance with applicable or relevant and appropriate requirements | 3 | The emission of some toxic air pollutants may be beyond the acceptable level (by law) for specific short periods during field activities. |  |
| Long-term effectiveness and permanence | 0 | This technique has almost no impact on decreasing the chance of future spontaneous combustion. |  |
| Reduction of toxicity, mobility, or volume through treatment | 0 | This technique does not decrease the volume or deactivate the spontaneous combustion source. |  |
| Short-term effectiveness | 5 | This technique has an immediate impact by extinguishing the fire. |  |
| Implement ability | 3 | Although this technique is not technically complicated, it is not easy to implement. It involves excellent planning and control to minimize the environmental and health impacts during the field activity. |  |
| Cost | 4 | 1.2 million USD; 1.66 years to implement with 8 backhoes, 2 bulldozers, 4 excavator dump trucks, and 2 water trucks with a high-pressure water spray | 1 USD = 30.17 THB |


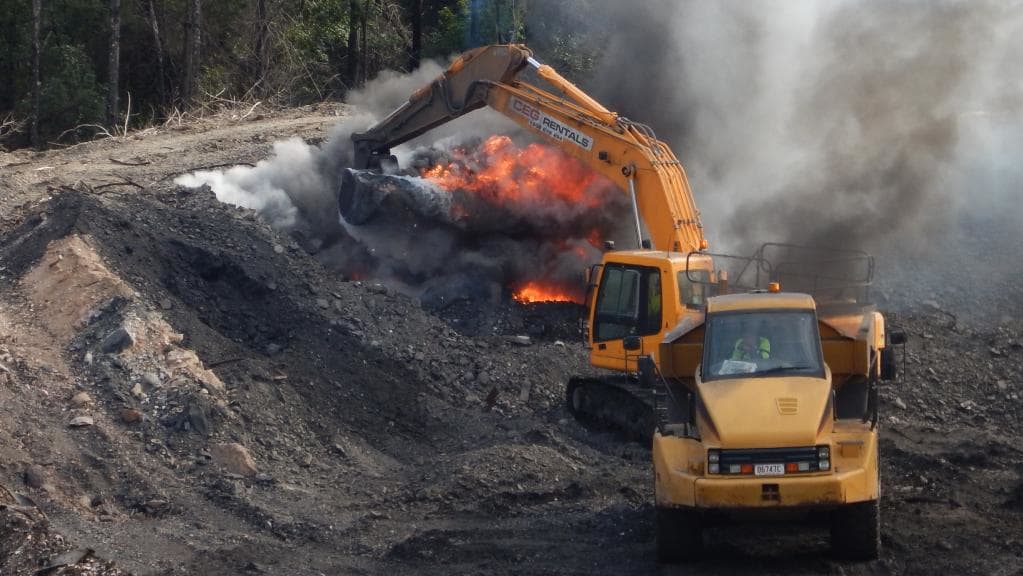


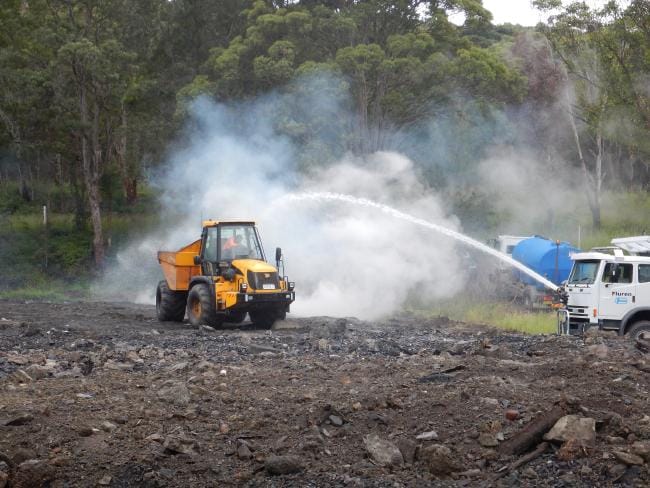


**Figure S10 Using excavation and water to extinguish current coal fire at Crangan Bay (The Daily Telegraph, 2014)**

1. **Surface sealing**

After the preparation step, surface sealing can be used to extinguish the fire passively. Surface sealing relies on creating an oxygen-deprived environment in the fire zone by isolation of the airflow path, i.e., keeping the newly diffused oxygen from entering the hot spot (Lui et al., 1998). This technique allows the existing oxygen in the hot spot to be eventually completely consumed. The spontaneous combustion will eventually stop in the absence of oxygen. The key is to completely seal the cracks and holes on both the waste heap and the ground surrounding the waste heap to ensure no new oxygen interacts with the hot spot or the waste pile. It consists of placing a thick “blanket” of compacted soil material over the affected area (see Figure S11). This soil layer can also attenuate the toxic gaseous emissions via sorption and chemical reaction at the solid–gas interface (Querol et al., 2011). Typically, clay-rich materials (1–2 m thick) with high water retention capacities will work effectively for this technique because the moisture content in clay reduces the voids between coal particles at the surface of the pile. In contrast, sandstone covers with low water retention need to be much thicker (5–10 m) (Sloss, 2015). The choice of geological material, either sand or clay, depends on its availability at the site. The thickness of surface sealing must be appropriate for the type of available geological materials. A reactive surface seal can also be used (Stracher et al., 2015). As shown in Figure S12, a layer of high-grade coal with low or no risk of spontaneous combustion (coal analysis is needed to ensure this property prior to usage) may be used as a reactive layer to completely consume oxygen in the air prior to entering the actual waste heap (Stracher et al., 2015). However, this reactive layer concept may not be cost-effective for a temporary fire extinguishment due to the price of high-grade coal and the frequency of spontaneous combustion, but it is a good candidate for long-term mine waste storage. Also, the selection of high-grade coal as a reactive layer must carefully consider its metal concentration and acid mine drainage potential to ensure that this application will cause no unintended environmental or health consequences.

Since extinguishing fires by surface sealing is a passive and gradual process, the decrease in temperature or gas emissions needs to be monitored to evaluate the progress of the procedure (Lu et al., 2017). Figure S13 illustrates a temperature sensor and a gas sensor implanted into a waste heap via drilling. The implanted sensors are more appropriate for long-term mine waste storage (as discussed below), while for existing fire suppression, temperature and gas can also be measured manually, as shown in Figure S13. The surface seal has been applied to stop spontaneous combustion at several sites (an example is shown in Figure S14 with installed temperature and gas monitoring).

If the surface sealing is designed to simply extinguish the existing fire, resistance to erosion and geotechnical instability may not be a concern because the soil layer will be used only temporarily. However, if the surface sealing technique is left to store the mine waste for extended periods, a good design, selection of material, and construction technique to provide resistance to erosion and geotechnical instability is imperative. Since this is a passive technique, one advantage over the excavation method is the lack of fire escalation and thus low or no risk of unintended nearby forest or range fires. Furthermore, this technique will not increase the release of toxic gaseous pollutants or particulate matter from the waste heap but gradually suppress emissions.

This method will work for the coal waste heap in this study since there are several cracks and holes on the waste heap that are responsible for the continuation of the spontaneous combustion. However, this technique would not be preferable if the ultimate mine waste management goal is to remove the waste for off-site disposal.


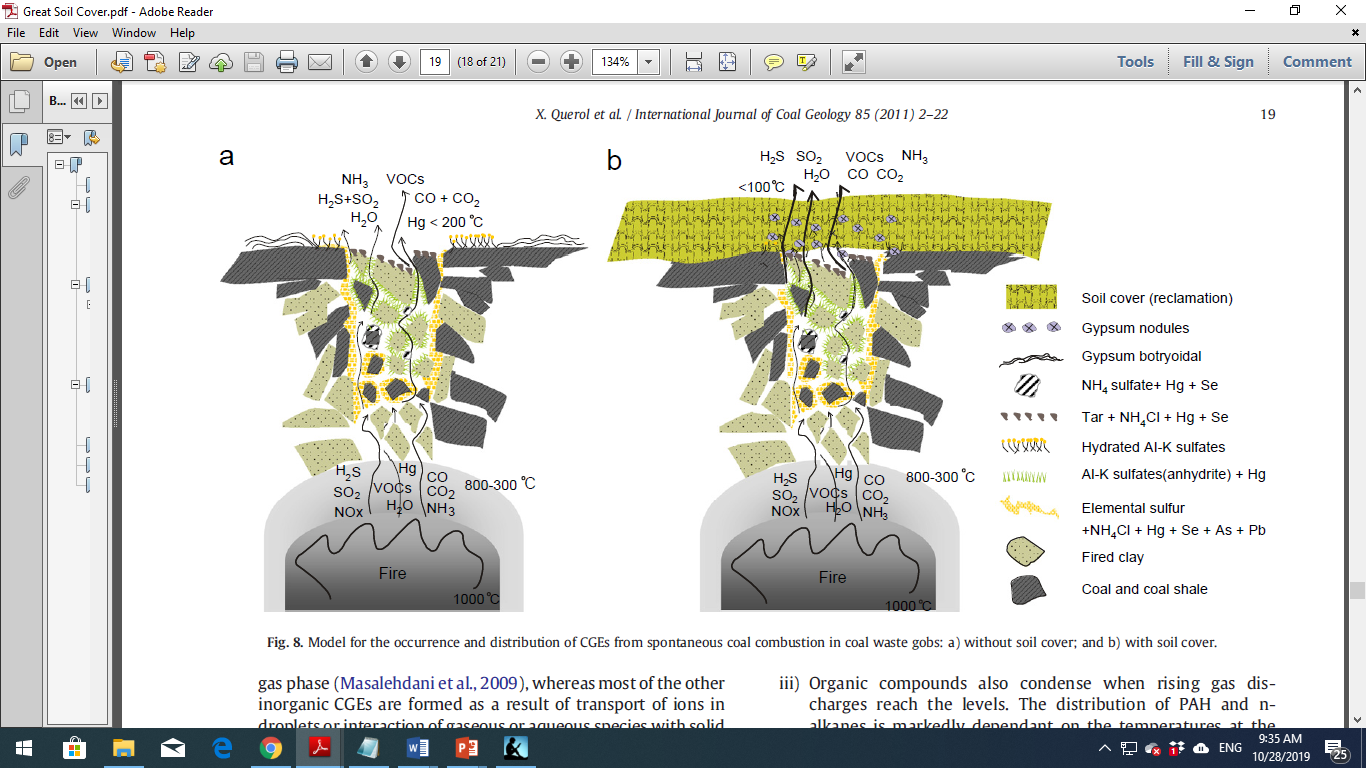


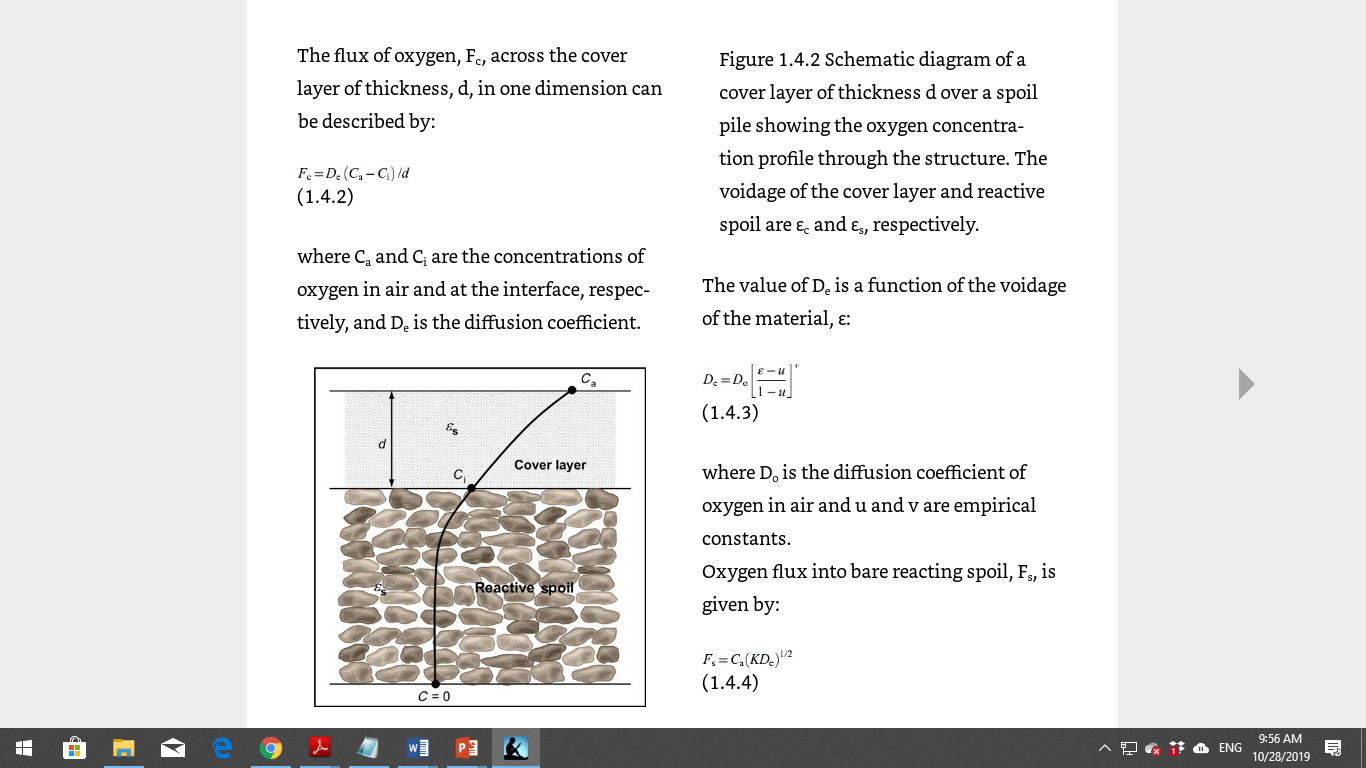
**Figure S11. Surface sealing technique to isolate the oxygen flow path, thereby stopping the fire. This surface seal layer will also attenuate the toxic gaseous emissions (Querol et al., 2011).**

**Figure S12. Reactive layer to completely deplete oxygen in the air (Stracher et al., 2015)**


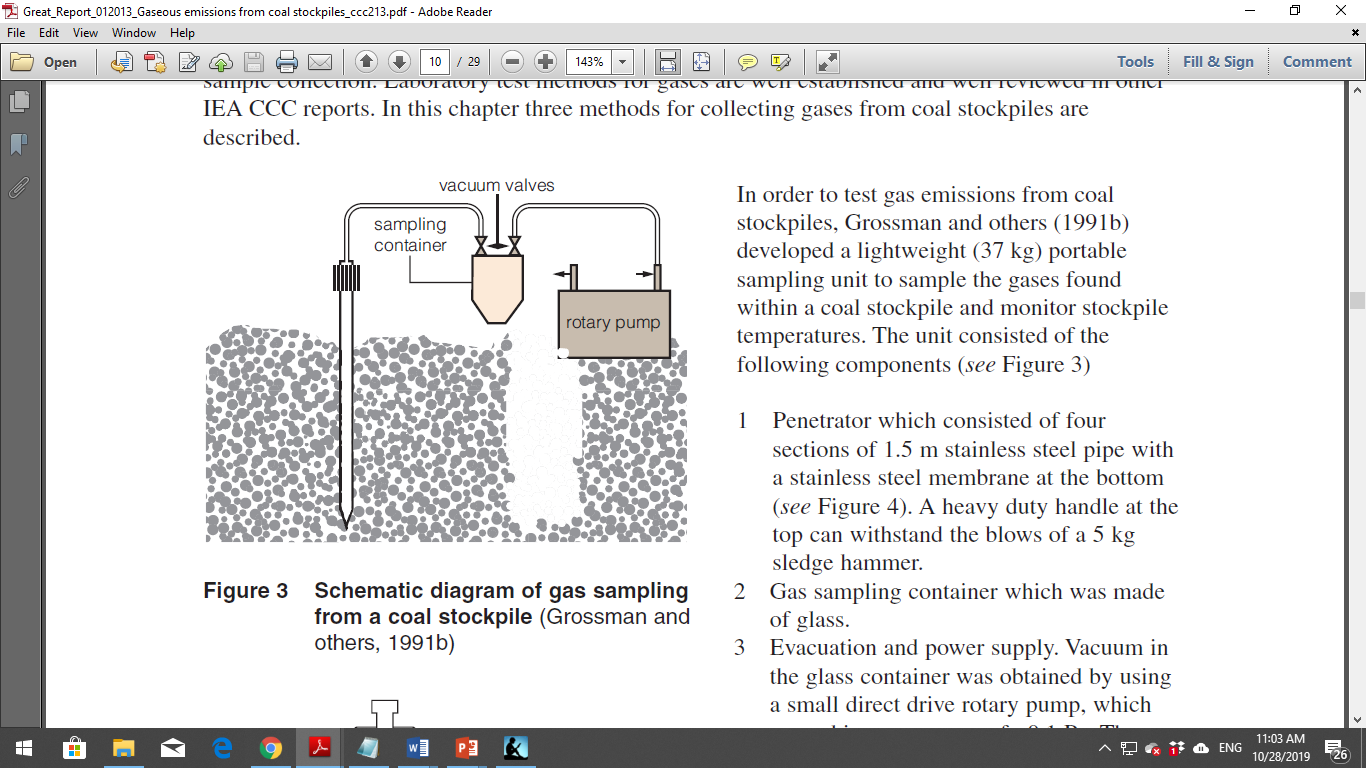

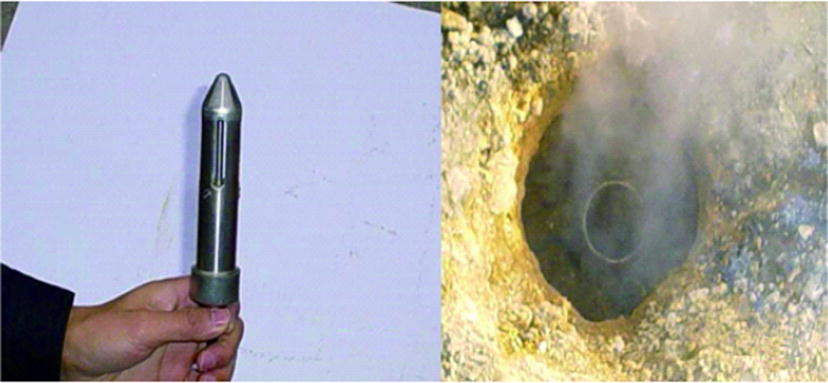


**Figure S13 Implanted temperature and gas sensor for monitoring the fire extinguishing progress (Lu et al., 2017; Zhang, 2013) and the manual monitoring process**


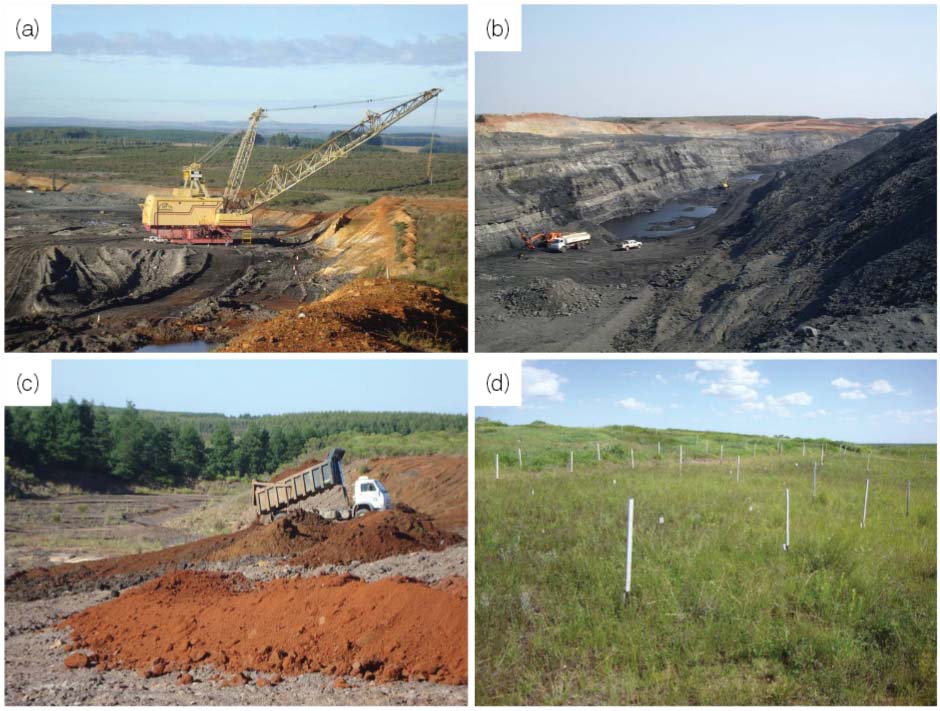


**Figure S14 (a-c) Application of surface seal on a coal waste heap (for long-term storage) and (d) implant monitoring system (Leal et al., 2016)**

Evaluation based on seven USEPA criteria

Based on the literature and the environmental condition of the study site, we assigned the following scores to this technique.

**Table S4 Evaluation of surface sealing technique based on seven USEPA criteria**

| **Criteria** | **Score Given** | **Rational** | **Note** |
| --- | --- | --- | --- |
| Overall protection of human health and the environment | 5 | Due to its passive nature, there will be no substantial release of toxic air pollutants during the fire suppression. |  |
| Compliance with applicable or relevant and appropriate requirements | 5 | Due to its passive nature, most of the time, the emission of some toxic air is unlikely beyond the value regulated by law. |  |
| Long-term effectiveness and permanence | 5 | The surface sealing is likely to suppress the future incidence of spontaneous combustion. |  |
| Reduction of toxicity, mobility, or volume through treatment | 0 | This technique does not decrease the amount or deactivate the spontaneous combustion source. |  |
| Short-term effectiveness | 0 | The surface sealing will slowly and passively extinguish the internal fire. |  |
| Implement ability | 3 | This technique is moderately challenging to implement. The soil layer must be well-designed as compaction is needed to achieve the goal. The type of available geological material to serve as the sealer may be scarce in the area, limiting the ease of implementation. |  |
| Cost | 2 | 3.0 million USD; 0.5 years to implement with 8 backhoes, 2 bulldozers, 4 excavator dump trucks, and 2 water trucks with a high-pressure water spray | 1 USD = 30.17 THB |

1. **Grouting or Injection of Inert Gas and Inhibitor**

The last approach evaluated for existing fire suppression is grouting or injection of inert gas and an inhibitor. When inert gases such as N_2_ and CO_2_ are injected into hot spots of the waste heap, the inert gas will replace O_2_ and prevent it from reaching the coal-mine waste due to positive pressure. Theoretically, this will suffocate and eventually stop the fire. However, practically, inert gases may leave the injection area by readily following an air leak from a preferential flow path (short circuit) instead of extinguishing the combusting hot spot as planned (Cheng et al., 2017). To be effective, surface sealing with a soil layer to eliminate all airflow leaks may be needed prior to the injection of the inert gas.

Grouting can also employ inhibitors, which can be liquid-based, gel-based, or foam-based. Conceptually, the basic grouting material is composed of water and solid particles or chemicals (ionic salts) combined with a foaming agent or macromolecule for foam-based or gel-based materials (Cheng et al., 2017; Cokaizzi, 2004; Lu et al., 2017). An effective grouting material must be heat resistant and highly flowable. It also needs to be capable of being applied directly to burning coal without causing a steam explosions or grout flashes (Cheng et al., 2017). Different grouting material components can achieve different fire suppression tasks. For example, once injected, the grouting material fills void spaces in the coal waste heap, effectively depleting the sources of oxygen. Next, water in the grouting material moistens the heated coal, which can reduce the heat of the mass. Solid particles or precipitates in the grouting materials can then cover and enclose the entire coal body to isolate the oxygen and extinguish the fire. If properly selected, the solid particles may also behave as a chemical inhibitor of coal oxidation by changing coal structures and lowering the tendency of coal to self-ignite at low temperatures (Cheng et al., 2017; Cokaizzi, 2004; Lu et al., 2017).

Liquid-based grouting materials can be a mixture of water and chemicals (ionic salts) such as sodium chloride, calcium carbonate, potassium chloride, lithium chloride, ferrous sulfate, ferric chlorate, or aluminum sulfate (up to 15% for best performance) and bentonite or clay to form an injectable slurry. Also, some liquid-based grouting material may be a mixture of clay and residuals (Lui et al., 1998), as shown in Table S5. The disadvantage of liquid-based grouting materials is their flowability. Upon injection, a grouting slurry is likely to flow downward due to gravity. Thus, the distribution may not be as good as a foam-based grouting agent, which expands and tends to follow the airflow path (Cheng et al., 2017; Cokaizzi, 2004). A foam-based material called Thermocell (Goodson & Associates, Inc.) is composed of sand, cement, water, a large amount of fly ash, and a foaming agent to produce a quantity of air-entraining foam (much like shaving cream) (Cokaizzi, 2004).

There are three kinds of gel-based grouting agents: gelatum, thickening gel, and composite gel. Gelatum is made of sodium silicate solution, coagulant (ammonium bicarbonate), fly ash, and water. The fraction of the silicate solution and coagulation is 5 to 10%. Thickening gel is composed of a thickening suspended agent (FCXF12, JXF1930), solid particles (sediment or fly ash), and water. Composite gel is made of gelatinizer (FHJ16, FCJ12), fly ash, and water. The ratio of solid particle to water is 1:1 to 2:1, and gelatinizer content only accounts for 0.06% of total water mass. This last kind of gel-based grouting material is the most widely used for fighting coal fires. Yet, gels have low fluidity and a small penetration range (Cheng et al., 2017).

Unlike excavation, the injection of inert gas and grouting material does not require heavy machines for earth work. Unlike surface sealing, the injection of inert gas and grouting material is an active technique that can directly manage the hot spot, making it much faster than the surface seal in fire elimination. This technique requires drilling of injection wells on the mine waste heap or into the surrounding ground. Thus, a survey to identify the depth and location of hot spots in the mine waste heap and the airflow path is critical to the efficacy of this technique. A survey of depth and location of hot spots in the mine waste heap can be determined by installing monitoring pipes to monitor temperature or gas emission (such as CO) (as shown in Figure S7). Moreover, the real-time delineation of hot spots can be done using a geoprobe equipped with a membrane interface probe (Figure S15) with a thermocouple sensor. Furthermore, a survey to identify the airflow path, especially from the ground leaking into the mine waste heap, can be accomplished by tracer gas injection. Figure S16 illustrates an example of using SF6 as a tracer gas to determine the airflow path from the ground that is connected to the mine waste heap (Zhai et al., 2017).


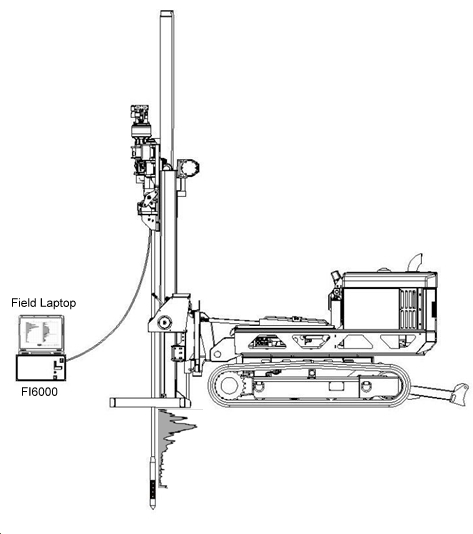


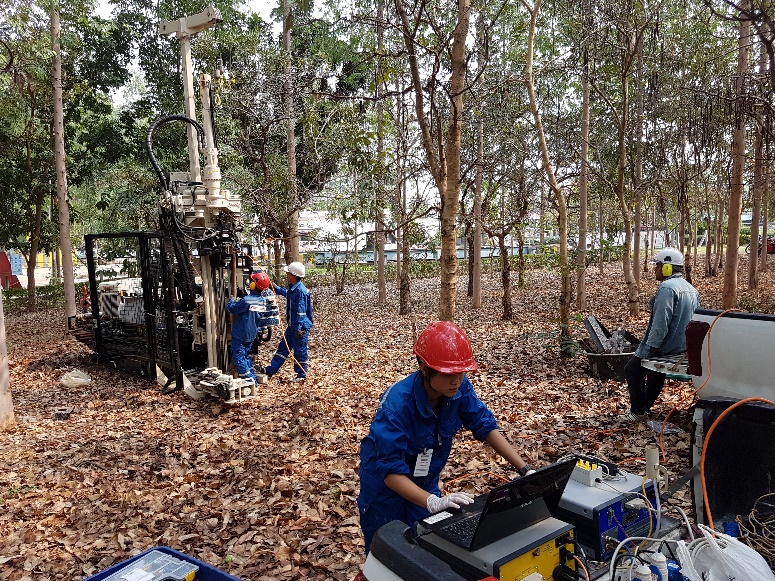


**Figure S15 Geoprobe equipped with a membrane interface probe with a thermocouple sensor for real-time delineation of hot spots.**


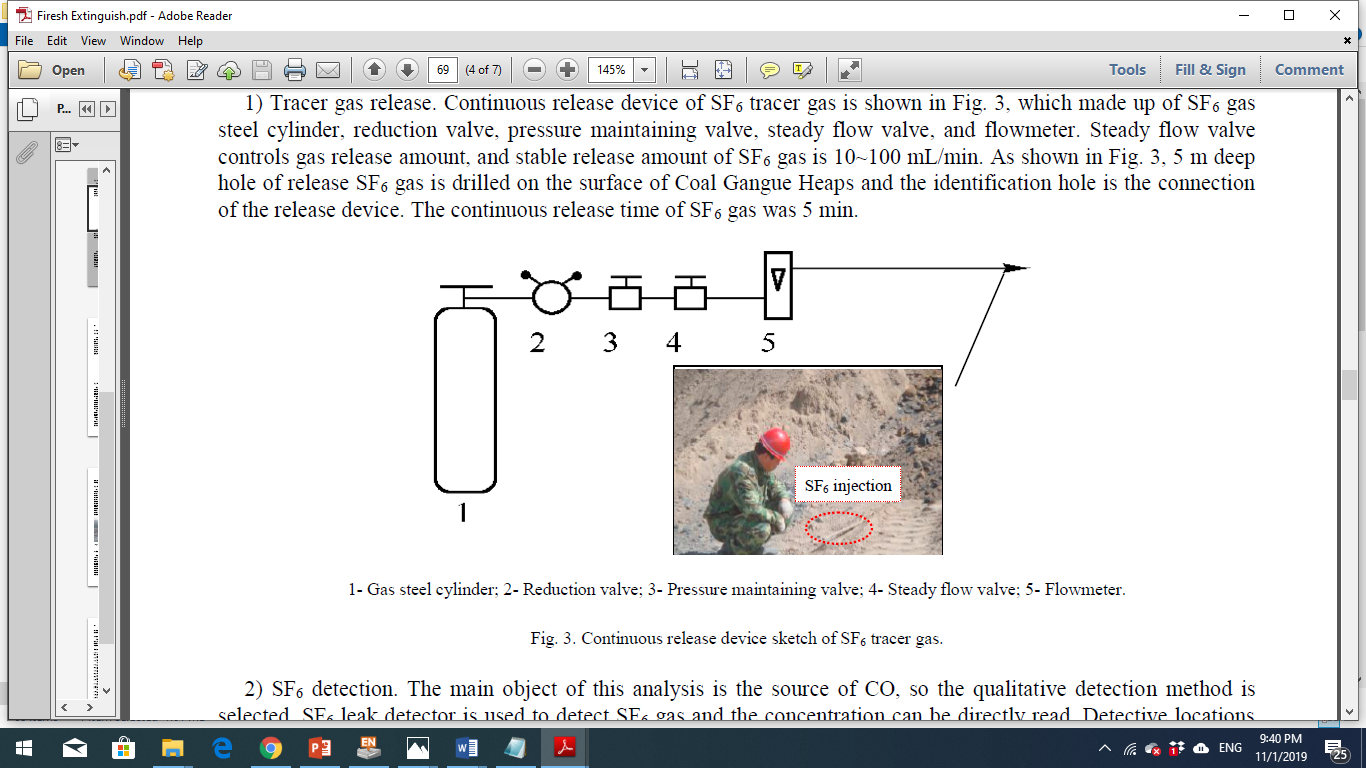


**Figure S16 A tracer test to determine the airflow path** **from the ground which is connected to the mine waste heap**


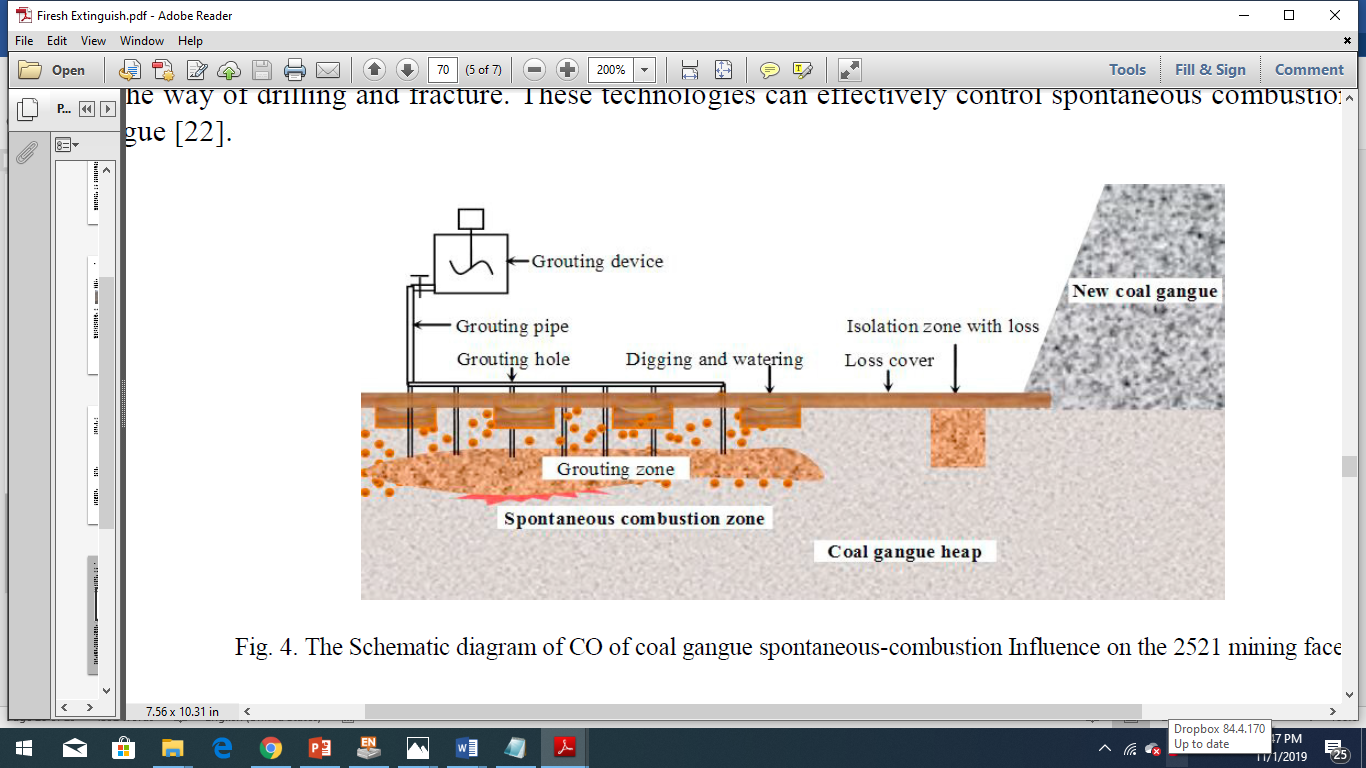


**Figure S17 Grouting system for injection of liquid-based materials**

Once the targeted hot spots are determined, injection wells can be installed, and grouting material can be injected. Figure S17 illustrates a successful injection of liquid-based grouting material to extinguish the fire deep inside a mine waste heap (Zhai et al., 2017). According to survey data of the distribution and area of the high-temperature zone of the mine waste heap, 47 holes (with a depth of 10 to 30 m) were drilled on the surface of the heap. The grouting material (volume =100,344 m^3^) was prepared on-site. As shown in Figure S18, the temperature of the hot spots greatly dropped, indicating the success of the operation. It should be noted that this site used grouting together with surface sealing (Zhai et al., 2017).


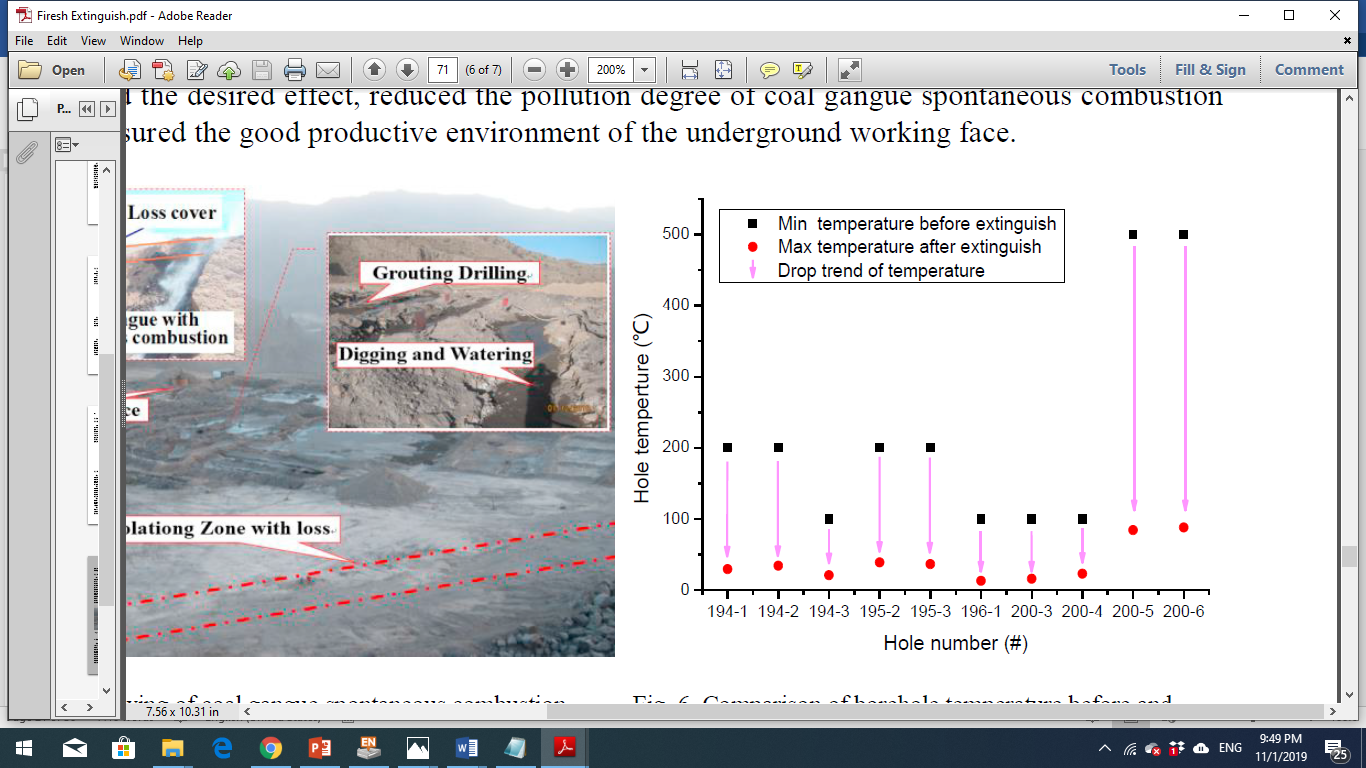


**Figure S18 Fire suppression efficacy of grouting procedure shown in Figure S10 (Zhai et al., 2017)**

**Table S5 Formulas of liquid grouting materials (Lui et al., 1998)**

| **Material** | **Concentration** | **Relative Viscosity** |
| --- | --- | --- |
| Lime+clay | 5+10% | 1.29 |
| Calcium carbide residual + coal ash | 5+10% | 1.02 |
| Calcium carbide residual | 15% | 1.17 |
| Clay | 15% | 1.13 |

Evaluation based on seven USEPA criteria

Based on the literature as well as the environmental condition of the study site, we assigned the following scores to this technique.

**Table S6 Evaluation of surface sealing based on seven USEPA criteria**

| **Criteria** | **Score Given** | **Rational** | **Note** |
| --- | --- | --- | --- |
| Overall protection of human health and the environment | 5 | Due to the nature of the grouting, no substantial release of toxic air pollutants occurs during the existing fire suppression. |  |
| Compliance with applicable or relevant and appropriate requirements | 5 | The emission of some toxic air is unlikely to exceed the regulated value by law most of the time. |  |
| Long-term effectiveness and permanence | 5 | The grouting technique appears to have a substantial residual effect on decreasing future spontaneous combustion |  |
| Reduction of toxicity, mobility, or volume through treatment | 3 | Grouting technique substantially inhibits the source of spontaneous combustion. |  |
| Short-term effectiveness | 5 | The grouting has an immediate impact on extinguishing spontaneous combustion. |  |
| Implement ability | 3 | This technique is moderately difficult to implement. It requires thorough site characterization to identify the hot spot for the grouting procedure. |  |
| Cost | 2 | 3.4 million USD; 0.6 years to implement with 10 grouting machines | 1 USD = 30.17 THB |

**Options for Coal-mine Waste Storage and Prevention of Spontaneous Combustion**

1. **No Action**

No action is an option that needs to be assessed according to USEPA remedial alternative consideration. For this specific mine waste heap, if no action is performed, spontaneous combustion will occur, causing the same health and environmental impacts discussed previously. This is unacceptable for the first two evaluation criteria and cannot be an appropriate remedial option. The negative impacts of no action will increase if the mining operations expand to include all (approx.) 600 acres of the mining area, almost 10 times the current waste heap area of 64 acres. This could increase waste generation, the potential for spontaneous combustion, and air pollution by an order of magnitude.

1. **No Action and Long-term Monitoring**

In this option, no action will be performed on the mine waste heap rather than installing a gas and temperature monitoring system (as shown in Figure S13) and preparing for emergency response. An emergency response protocol must be established. According to Zhu et al. (2018), if the temperature inside the heap (at the hot spot) is 55–77˚C, no action is needed but strengthening the monitoring of the spontaneous coal combustion. When the temperature is greater than 90˚C, emergency response, including analyzing air leakage and injection of grouting materials to localize the fire zone, will be performed. Once the temperature is greater than 100–115˚C, more intensive emergency response is necessary, including drilling and injection of anti-fire materials into the fire zone to seal it (Zhu et al., 2018). Other emergency response protocols may be applied, depending on the specific risk management team. This is the cheapest option for a short-term investment but risks long term liability and incurs a relatively high cost of monitoring and emergency response. Also, if the monitoring and preventive measures are not sufficient, the spontaneous combustion may occur, releasing toxic gaseous pollutants to the nearby villages. Noticeably, the emergency response steps discussed above are performed to prevent the formation of spontaneous combustion hot spots inside the waste heap (Zhu et al., 2018). These steps do not prohibit low-temperature oxidation at the surface of the heap.

Evaluation based on seven USEPA criteria

Based on the literature and the environmental conditions of the study site, we assigned the following scores to this technique.

**Table S7 Evaluation of no action and long-term monitoring by seven USEPA criteria**

| **Criteria** | **Score Given** | **Rational** | **Note** |
| --- | --- | --- | --- |
| Overall protection of human health and the environment | 1 | Due to the challenge of practical monitoring and emergency response, the spontaneous combustion may occasionally release toxic gas to unacceptable levels |  |
| Compliance with applicable or relevant and appropriate requirements | 3 | The emission of some toxic air may occasionally be beyond the regulated value by law |  |
| Long-term effectiveness and permanence | 1 | Long-term monitoring and emergency response is very challenging |  |
| Reduction of toxicity, mobility, or volume through treatment | 0 | No treatment of coal to inhibit the source of spontaneous combustion. |  |
| Short-term effectiveness | 0 | No action to establish proper storage conditions for mine waste as only providing monitoring and emergency response |  |
| Implement ability | 0 | Difficult to implement; several technical challenges; involve significant risk during implementation. The mine personnel would have no experience of successful emergency response to spontaneous combustion. |  |
| Cost | 3 | 10 million USD; for 10 years of monitoring and emergency response | 1 USD = 30.17 THB |

1. **On-site Storage with Surface Sealing and Long-term Monitoring**

This option is similar to using surface seal to passively extinguish a fire, except that the surface seal must be designed for long-term storage of the mine waste. Thus, erosion control and slope stability of the surface seal and the mine waste heap must be properly designed. For this reason, the mine waste heap should be separated into two small heaps for slope stability and erosion prevention. Both temperature and gas monitoring systems must be installed to monitor the performance of the surface seal and the safety of the storage. Vegetation should be planted in the mine waste heap (see Figure S14). Seasonal maintenance of the surface seal must be planned, and emergency response must be prepared for unexpected situations. This management option still risks long-term liability. Moreover, this option does not guarantee the prevention of leaching of metals and acid mine drainage from the mine waste heap.

Evaluation based on seven USEPA criteria

Based on the literature and the suitability with the environmental condition of the site in the present study, we gave the following scores to this technique.

**Table S8 Evaluation of On-site Storage with Surface Sealing and Long-term Monitoring**

| **Criteria** | **Score Given** | **Rational** | **Note** |
| --- | --- | --- | --- |
| Overall protection of human health and the environment | 3 | Acid mine drainage may occur but at a much lower level. Thus, there may be less leaching of metals. |  |
| Compliance with applicable or relevant and appropriate requirements | 4 | Due to its preventive nature, the emission of some toxic air is unlikely beyond the regulated value by law most of the time. Some acid mine drainage may occur but should be mild with appropriate surface sealing. |  |
| Long-term effectiveness and permanence | 4 | The surface sealing is likely to prevent future spontaneous combustion during storage |  |
| Reduction of toxicity, mobility, or volume through treatment | 0 | This technique does not treat the coal waste but just prohibits its interaction with oxygen |  |
| Short-term effectiveness | 0 | The surface sealing will slowly and passively provide suitable conditions for mine waste storage |  |
| Implement ability | 3 | This technique is moderately difficult to implement. Appropriate design of the soil layer and compaction is needed to achieve the goal. |  |
| Cost | 3 | 7.9 million USD; for 10 years of monitoring and emergency response | 1 USD = 30.17 THB |

1. **On-site Secure Landfill and Long-term Monitoring**

In this option, the mine waste heap will be stored in a secure landfill (Figure S19), which is a standard way to dispose of hazardous waste. With appropriate long-term monitoring of the leachate collection system, neither metal leaching nor acid mine drainage is supposed to occur. The landfill has two layers of a leachate collection system to prohibit any leakage. This option is very expensive and requires permission to use the land to construct a secure landfill in the forest of the Ban Chang area.


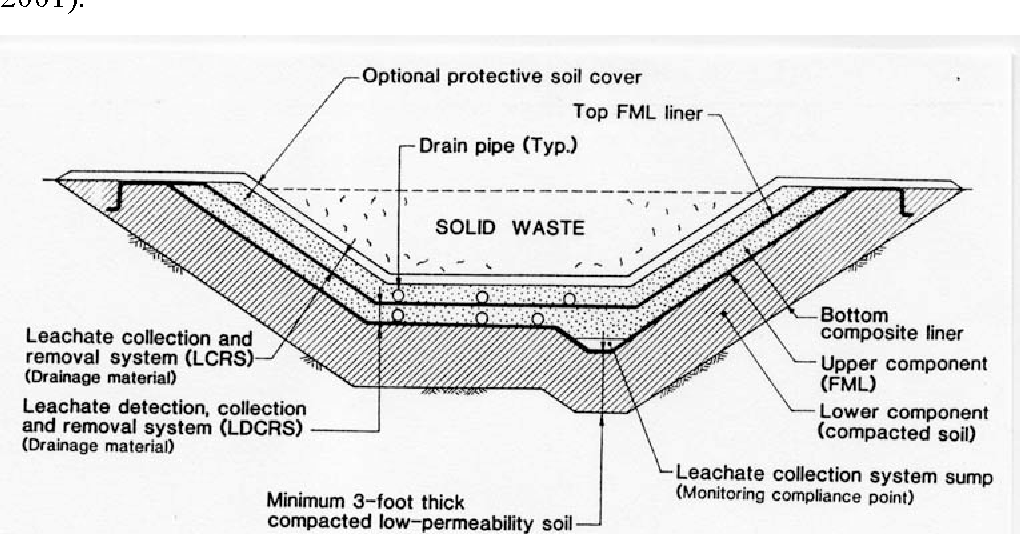


**Figure S19 A typical secure landfill for hazardous waste disposal**

Evaluation based on seven USEPA criteria

Based on the literature and the environmental condition of the study site, we assigned the following scores to this technique.

**Table S9 Evaluation of On-site Secure Landfill and Long-term Monitoring**

| **Criteria** | **Score Given** | **Rational** | **Note** |
| --- | --- | --- | --- |
| Overall protection of human health and the environment | 5 | With appropriate design, nothing can be released from a secure landfill |  |
| Compliance with applicable or relevant and appropriate requirements | 5 | No substantial emission or leachate. This should comply with existing laws. |  |
| Long-term effectiveness and permanence | 5 | Elimination of long-term potential spontaneous combustion and water contamination |  |
| Reduction of toxicity, mobility, or volume through treatment | 0 | This technique does not treat the coal waste but just prohibits its interaction with oxygen |  |
| Short-term effectiveness | 3 | It takes some time to build a secure landfill and more time to remove the mine waste to the landfill for proper disposal |  |
| Implement ability | 3 | This technique is moderately difficult to implement. It is not easy to build an adequate and secure landfill in the middle of the forest. Also, siting and geological condition must be favorable for landfill use (i.e., low soil permeability and the high interval between the bottom of the landfill and the shallow groundwater table) |  |
| Cost | 0 | 135 million USD; for 10 years of monitoring and emergency response | 1 USD = 30.17 THB |

1. **On-site Storage in Mine Lake and Long-term Monitoring**

One option is to transport all the mine waste to the mine lake close to the coal mine for storage. Since this lake is full of water all year long, it should have enough water to submerge the mine waste to prevent spontaneous combustion. This is technically feasible since if the coal waste is more than 40–50% saturated with water, no spontaneous combustion takes place. Nevertheless, this option will result in leaching of metals from the coal waste as well as acid mine drainage, subsequently causing surface water and groundwater contamination. Thus, this will seriously violate the first two criteria and should not be considered a viable option.

Evaluation based on seven USEPA criteria

Based on the literature and as the environmental condition of the study site, we assigned the following scores to this technique.

**Table S10 Evaluation of On-site Storage in Mine Lake and Long-term Monitoring**

| **Criteria** | **Score Given** | **Rational** | **Note** |
| --- | --- | --- | --- |
| Overall protection of human health and the environment | 0 | Since the mine waste is soaked with water all the time, acid mine drainage is likely to be aggressive, causing severe water and groundwater contamination. |  |
| Compliance with applicable or relevant and appropriate requirements | 0 | There is a risk that acid mine drainage and water contamination can be greater than the acceptable legal level |  |
| Long-term effectiveness and permanence | 1 | High potential for water contamination due to acid mine drainage |  |
| Reduction of toxicity, mobility, or volume through treatment | 0 | This technique does not treat the coal waste but stores it in a lake |  |
| Short-term effectiveness | 0 | Immediate and high potential for water contamination due to acid mine drainage |  |
| Implement ability | 0 | Difficult to implement; several technical challenges; involves significant risk of acid mine drainage during implementation |  |
| Cost | 5 | 1.4 million USD | 1 USD = 30.17 THB |

1. **Off-site Management: Disposal in Secure Landfill or Waste to Energy**

The most expensive option (in the short-term) with the least liability (long-term) is off-site management. All coal-mine waste, as well as contaminated soil, will be excavated and removed to manage off-site. The contaminated soil includes acid mine drainage precipitates, which will be deposited into the natural waterway and onto agricultural land. Two options of off-site management are available for coal-mine waste. First, the coal waste may be disposed of in a secure landfill due to its spontaneous combustion potential. Second, the waste can be used as an alternative fuel in a rotary kiln for cement production. Although these two options may be similarly expensive, using mine waste for the waste-to-energy process is more beneficial from a circular economy perspective. Thailand has both secure landfills and waste-to-energy plants. For this reason, if Myanmar is not ready for the off-site management of the mine waste, the waste can be transported to Thailand.

Evaluation based on seven USEPA criteria

Based on the literature and the environmental condition of the study site, we assigned the following scores to this technique.

**Table S11 Off-site Management: Disposal in Secure Landfill or Waste to Energy**

| **Criteria** | **Score Given** | **Rational** | **Note** |
| --- | --- | --- | --- |
| Overall protection of human health and the environment | 5 | Off-site management does not result in the release of toxic gas or water contaminants during storage |  |
| Compliance with applicable or relevant and appropriate requirements | 5 | Off-site management does not result in the release of toxic gas or water contaminants during storage |  |
| Long-term effectiveness and permanence | 5 | Elimination of long-term potential spontaneous combustion and water contamination |  |
| Reduction of toxicity, mobility, or volume through treatment | 5 | Complete removal of mine waste from the area |  |
| Short-term effectiveness | 5 | The waste is removed immediately |  |
| Implement ability | 5 | Off-site management for a secure landfill or waste-to-energy has been used to manage hazardous waste throughout the world. Logistics of coal-mine waste is possible in the same way that the mine transports coal out of the mine for commercial purposes. |  |
| Cost | 0 | 293 million USD | 1 USD = 30.17 THB |


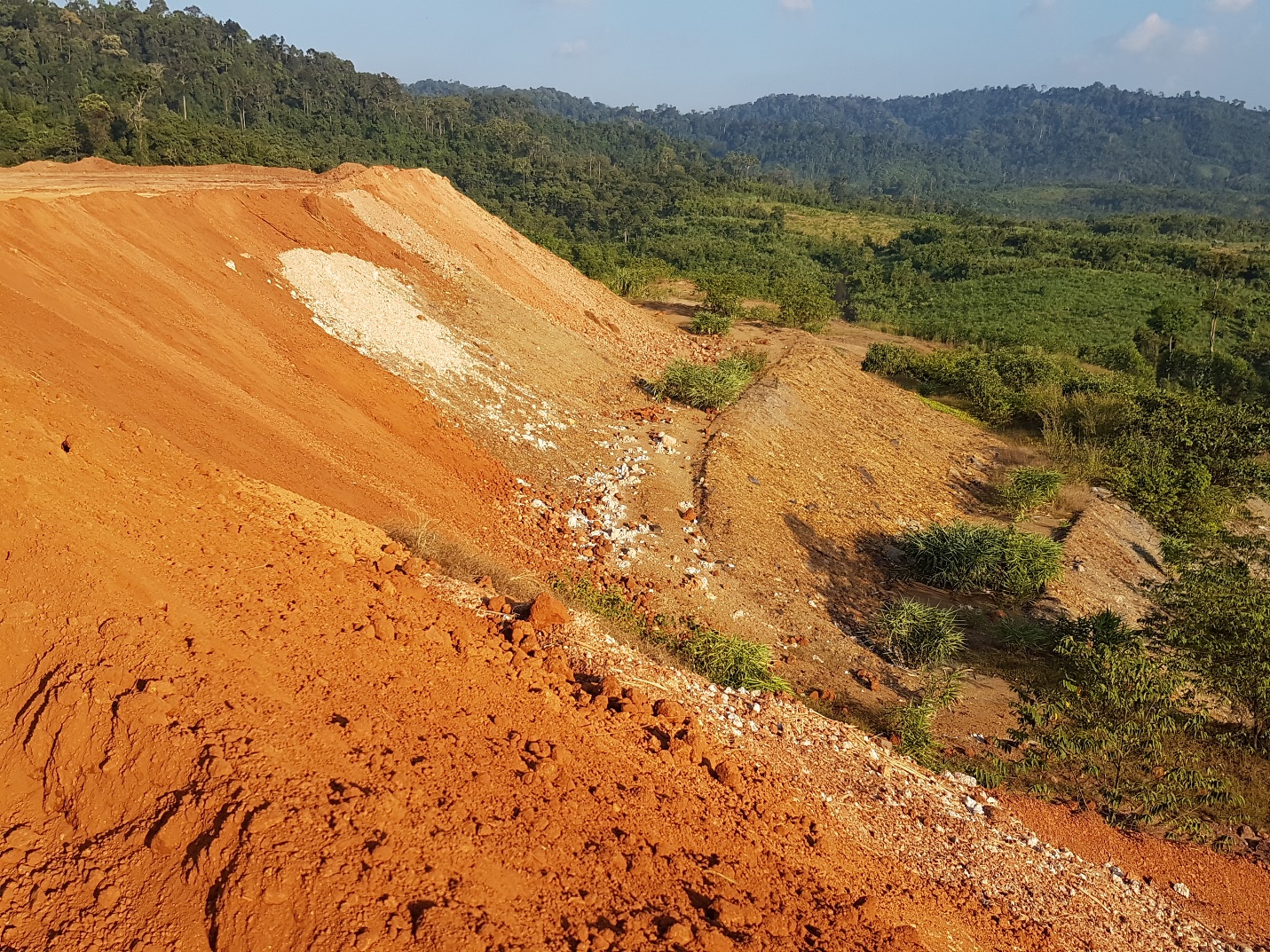


**Figure S20 The procedures currently performed by the mine without any public participation are surface sealing using sandy clay (2 m thick) for existing fire suppression followed by on-site storage of the mine waste with the same surface sealing but without any long-term monitoring**


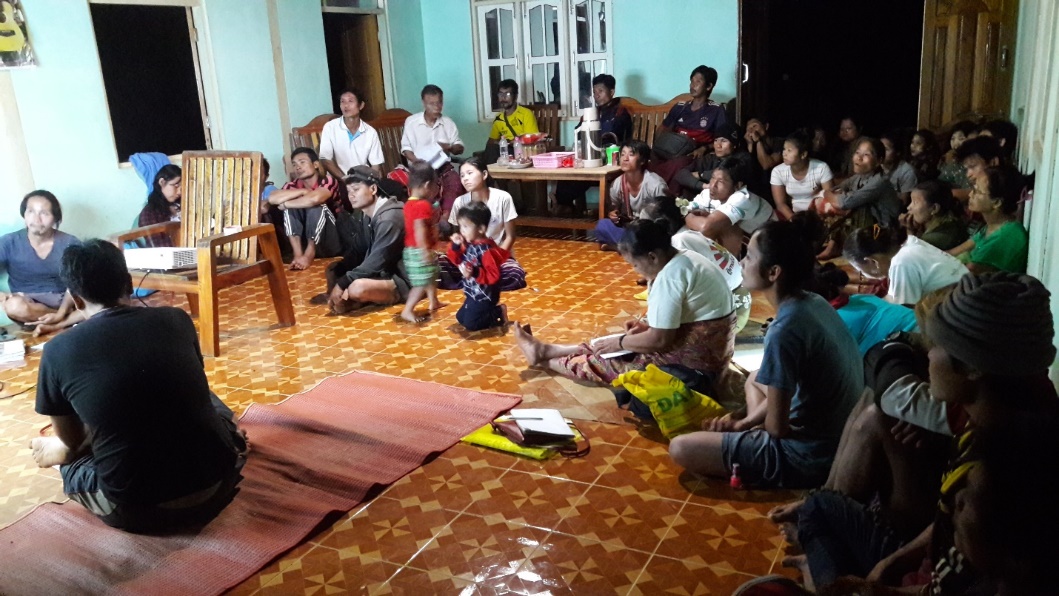


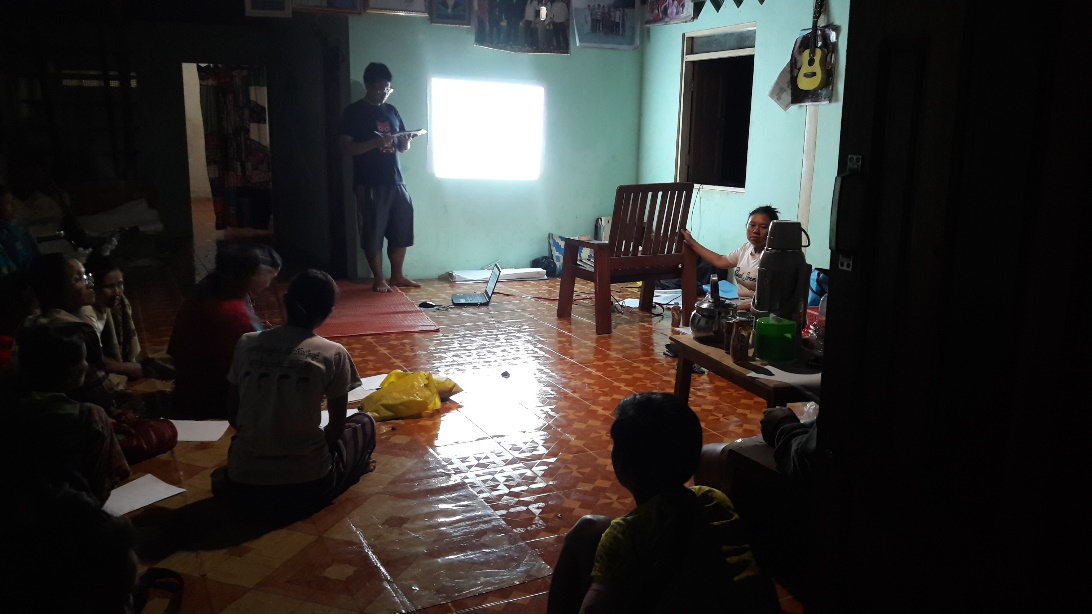


**Figure S21 Meeting with villagers to present the available management options for existing fire suppression and proper coal-mine waste storage as well as their evaluation based on the USEPA’s seven criteria for remediation selection strategies**

**Reference**

Cheng, W., Hu, X., Xie, J., Zhao, Y., 2017. An intelligent gel designed to control the spontaneous combustion of coal: Fire prevention and extinguishing properties. Fuel 210, 826-835.

Cokaizzi, G.J., 2004. Prevention, control and/or extinguishment of coal seam fires using cellular grout. Int. J. Coal Geol. 59, 75-81.

Leal, O.d.A., Castilhos, R.M.V., Pinto, L.F.S., Pauletto, E.A., Lemes, E.S., Kunde, R.J., 2016. Initial Recovery of Organic Matter of a Grass-Covered Constructed Soil after Coal Mining. Rev. Bras. Ciênc. Solo 40, e0150384.

Lu, W., Cao, Y.-J., Tien, J.C., 2017. Method for prevention and control of spontaneous combustion of coal seam and its application in mining field. Int. J. Min. Sci. Technol. 27, 839-846.

Lui, C., Li, S., Qiao, Q., Wang, J., Pan, Z., 1998. Management of Spontaneous Combustion in Coal Mine Waste Tips in China. Water Air Soil Poll. 103, 441-444.

Querol, X., Zhuang, X., Font, O., Izquierdo, M., Alastuey, A., Castro, I., van Drooge, B.L., Moreno, T., Grimalt, J.O., Elvira, J., Cabañas, M., Bartroli, R., Hower, J.C., Ayora, C., Plana, F., López-Soler, A., 2011. Influence of soil cover on reducing the environmental impact of spontaneous coal combustion in coal waste gobs: A review and new experimental data. Int. J. Coal Geol. 85, 2-22.

Sloss, L.L., 2015. Assessing and managing spontaneous combustion of coal. IEA Clean Coal Centre, London, p. 55.

Song, Z., Kuenzer, C., 2014. Coal fires in China over the last decade: A comprehensive review. Int. J. Coal Geol. 133, 72-99.

Stracher, G.B., Prakash, A., Sokol, E.V., 2015. Coal and Peat Fires: A Global Perspective: Volume 3: Case Studies – Coal Fires. Elsevier Science, Amsterdam.

The Daily Telegraph, 2014. Crangan Bay coal fire finally out after authorities spend four months digging for solution. <https://www.dailytelegraph.com.au/newslocal/central-coast/crangan-bay-coal-fire-finally-out-after-authorities-spend-four-months-digging-for-solution/news-story/4cc27ff97b9ea8b015c0b7665685ff7d>.

United States Environmental Protection Agency, 1990. A Guide to Selecting Superfund Remedial Actions. US.EPA, Washington DC, p. 6.

Zhai, X., Wu, S., Wang, K., Drebenstedt, C., Zhao, J., 2017. Environment influences and extinguish technology of spontaneous combustion of coal gangue heap of Baijigou coal mine in China. Energy Procedia 136, 66-72.

Zhang, X., 2013. Gaseous emissions from coal stockpiles. IEA Clean Coal Centre, p. 29.

Zhu, H., Sheng, K., Zhang, Y., Fang, S., Wu, Y., 2018. The stage analysis and countermeasures of coal spontaneous combustion based on “five stages” division. PLoS One 13, e0202724.
